# mRNA cancer vaccines from bench to bedside: a new era in cancer immunotherapy

**DOI:** 10.1186/s40364-024-00692-9

**Published:** 2024-12-18

**Authors:** Alireza Shariati, Pouria Khani, Farzad Nasri, Hamed Afkhami, Arya Khezrpour, Sina Kamrani, Fatemeh Shariati, Sajad Alavimanesh, Mohammad Hossein Modarressi

**Affiliations:** 1 School of Medicine, Tehran University of Medical Sciences (TUMS), Tehran, Iran; 2 Department of Medical Genetics, School of Medicine, Tehran University of Medical Sciences (TUMS), Tehran, Iran; 3 Department of Immunology, School of Medicine, Iran University of Medical Sciences, Tehran, Iran; 4 Cellular and Molecular Research Center, Qom University of Medical Sciences, Qom, Iran; 5 Nervous System Stem Cells Research Center, Semnan University of Medical Sciences, Semnan, Iran; 6 Department of Medical Microbiology, Faculty of Medicine, Shahed University, Tehran, Iran; 7 Department of Orthopedic, Faculty of Medicine, Guilan University of Medical Sciences, Rasht, Iran; 8 Department of Genetics, North Tehran Branch, Islamic Azad University, Tehran, Iran; 9 Student Research Committee, Shahrekord University of Medical Sciences, Shahrekord, Iran; 10 Cellular and Molecular Research Center, Basic Health Sciences Institute, Shahrekord University of Medical Sciences, Shahrekord, Iran

**Keywords:** Cancer, mRNA, Vaccine, mRNA cancer vaccine, Cancer treatment, Cancer immunotherapy

## Abstract

Harnessing the power of the immune system to target cancer cells is one of the most appealing approaches for cancer therapy. Among these immunotherapies, messenger ribonucleic acid (mRNA) cancer vaccines are worthy of consideration, as they have demonstrated promising results in clinical trials. These vaccines have proven to be safe and well-tolerated. They can be easily mass-produced in a relatively short time and induce a systemic immune response effective against both the primary tumor and metastases. Transcripts encoding immunomodulatory molecules can also be incorporated into the mRNA, enhancing its efficacy. On the other hand, there are some challenges associated with their application, including mRNA instability, insufficient uptake by immune cells, and intrinsic immunogenicity, which can block mRNA translation. Many innovations have been suggested to overcome these obstacles, including structural modification (such as 5’ cap modification), optimizing delivery vehicles (especially dendritic cells (DCs) and nanoparticles), and using antigens that can enhance immunogenicity by circumventing tolerance mechanisms. A popular approach is to combine mRNA cancer vaccines with traditional and novel cancer treatments like chemotherapy, radiotherapy, and immune checkpoint blockade (ICB). They are most efficacious when combined with other therapies like ICBs. There is still a long way to go before these vaccines enter the standard of care for cancer patients, but with the incredible pace of development in this field, their clinical application will soon be witnessed. This review highlights the recent advances and challenges of mRNA cancer vaccines. Finally, some of the most prominent clinical applications of these vaccines will be reviewed.

## Introduction and principles

Since 1796, the year Edward Jenner first demonstrated the viability of the *Variolae* vaccine for inducing immunity against smallpox, vaccines have saved millions of lives from a plethora of infectious micro-organisms. Nowadays, vaccines look very different from their predecessors. They are no longer designed just to target infectious agents, but also a wide variety of other human disorders, most prominently cancer [2, 3]. Although most cancer vaccines are currently in clinical and preclinical stages, hopefully, they can soon become potent tools to aid clinicians in treating different malignancies. Some of these vaccines have already been integrated into the standard of care for some malignancies, most notably Sipuleucel-T for prostate cancer [4].

These cancer vaccines can be classified into four categories: cell-based vaccines including DC vaccines and tumor-cell vaccines, nucleic-acid-based vaccines including mRNA and DNA vaccines, protein/peptide-based vaccines and viral-based vaccines, although compartmentalization in this fashion is not quite accurate as many of these vaccines can be combined. For example, many mRNA vaccines use DCs as vectors.

Immunization against malignancies through mRNA cancer vaccines is a hot topic in cancer immunotherapy. Messenger ribonucleic acid molecules provide a template for synthesizing virtually any protein, translated once inside the cytoplasm. Since they provide a significant but transient expression, they can be utilized in many etiologically different disorders, most notably infectious diseases and cancer [5, 6].

The Moderna mRNA-1273 and Pfizer-BioNTech BNT162B2 COVID-19 vaccines are the most eminent examples of mRNA vaccine employment in infectious diseases, contributing to the plummet of COVID-19’s burden worldwide [7]. This successful experience paved the way for the future development of other mRNA-based vaccines targeting other forms of diseases as well, especially cancer. Despite numerous advantages compared to conventional vaccines, during COVID-19 vaccination, mRNA vaccines displayed some downsides, such as local (injection site) side effects, systemic side effects, and storage complications requiring an ultralow temperature for preservation [8–10].

The basic rationale behind mRNA-based cancer vaccines is simple: To deliver TA-encoding mRNA such as those that encode cancer/testis antigens (CTAs) into host cells, especially antigen-presenting cells (APCs) [11]. Once inside the cell’s cytoplasm, the mRNA is translated and TAs are biosynthesized. Subsequently, they are loaded on major histocompatibility complexes (MHCs) to be presented to T-cells, priming the immune system for targeting tumor cells. They can activate both humoral and cellular immune responses [12]. Despite subtle differences, MHC I and II are quite structurally similar. MHC I is expressed on the surface of each nucleated cell in the body and presents antigens to cytotoxic CD8 + T-cells, thus it is more associated with the cellular immune response. It is responsible for the introduction of antigens that are processed in the endosomal system. However, MHC II is primarily expressed on APCs and presents antigens to CD4 + helper T-cells and it is more intensely involved in the development of a humoral immune response. It is responsible for the presentation of endogenous proteins (those expressed by our genes) [13].

Similar to DNA vaccines, they can simultaneously deliver multiple tumor antigens (TAs). Unlike peptide vaccines, they are suited for encoding full-length TAs with several epitopes, broadening the resulting immune response [14]. They elicit an extensive T-cell response resulting from the activation of both CD4 + and CD8 + T-cells. They are also relatively safe compared to viral vaccines, posing no infectious risk [15–18].

The main component of the anti-tumor immunity is the cellular CD8 + cytotoxic T-cell response. It is responsible for directly targeting tumor cells following the recognition of their surface markers. On the other hand, the anti-tumor cellular immune response is paramount for the function of ICBs. ICBs work by preventing the inhibitory effect of tumors on the immune cells of the tumor microenvironment (TME). In order for ICBs to be efficacious, clones of tumor-reactive CD8 + cytotoxic T-cells should exist in the patient’s body. This makes mRNA cancer vaccines (especially those that encode neo-antigens or TSAs) and ICBs a good combination therapy for cancer patients.

A production method currently being applied to synthesize self-amplifying mRNA (SAM) and non-replicating mRNA (nrRNA) vaccines is called in vitro transcription (IVT) [19, 20]. This cell-free method is replacing other means of mRNA mass-production including cell-based methods, as it provides a simpler, faster, and cleaner large-scale production. This approach exploits a bacteriophage-derived DNA-dependent RNA-polymerase such as SP6, T3, or T7 and a linearized DNA template that contains the tumor-antigen(s) of interest and sometimes adjuvants co-encoded in the same platform [21]. Other than functional antigen-encoding mRNA, other mRNA species such as dsRNA and hairpins are also formed during IVT, which could harm the vaccine’s efficacy. These species are usually removed from the mixture before administration. Purification techniques are mentioned later on in this paper. In this article, mRNA cancer vaccines are going to be discussed in more detail as they are stealing the spotlight, especially after the huge success of mRNA-based COVID-19 vaccines which garnered the attention of researchers and the public toward a potentially life-saving platform of cancer therapeutics [22].

Despite the advantages of mRNA cancer vaccines, there are some challenges that hinder their widespread application in clinical settings. Firstly, current trials have shown that mRNA cancer vaccines are not potent enough to elicit a response that will drastically change patients’ outcomes. Secondly, these vaccines are rather expensive to produce, store, and transport since they mRNA molecules are relatively unstable. Also, the process of mRNA vaccine production (especially patient-specific vaccines) is time-consuming which limits their applicability for treating cancer patients [23]. In this paper, recent approaches to address these shortcoming will be thoroughly discussed.

It should be mentioned that throughout this article there are many instances in which vaccines containing mRNA-transfected DCs have been categorized as mRNA vaccines. We believe that these kinds of vaccines can be considered DC vaccines and mRNA vaccines at the same time and hence, we have included them in our paper.

## Classification of mRNA vaccine platforms

mRNA cancer vaccine platforms can be divided into three categories: non-replicating mRNA (nrRNA), SAM, and trans-amplifying mRNA (taRNA).

### Non-replicating mRNA (nrRNA)

Also called conventional mRNA vaccination, this platform utilizes simple mRNA molecules containing usual eukaryotic mRNA segments including 5’ Cap, 3’ and 5’ untranslated regions, poly(A) tail, and one or more open reading frame(s). The major advantage of this approach is simplicity and relative ease of production compared to other mRNA vaccine classes. This simplicity also causes limited activity and stability [1].

### Self-amplifying mRNA (SAM)

**Fig. 1:**
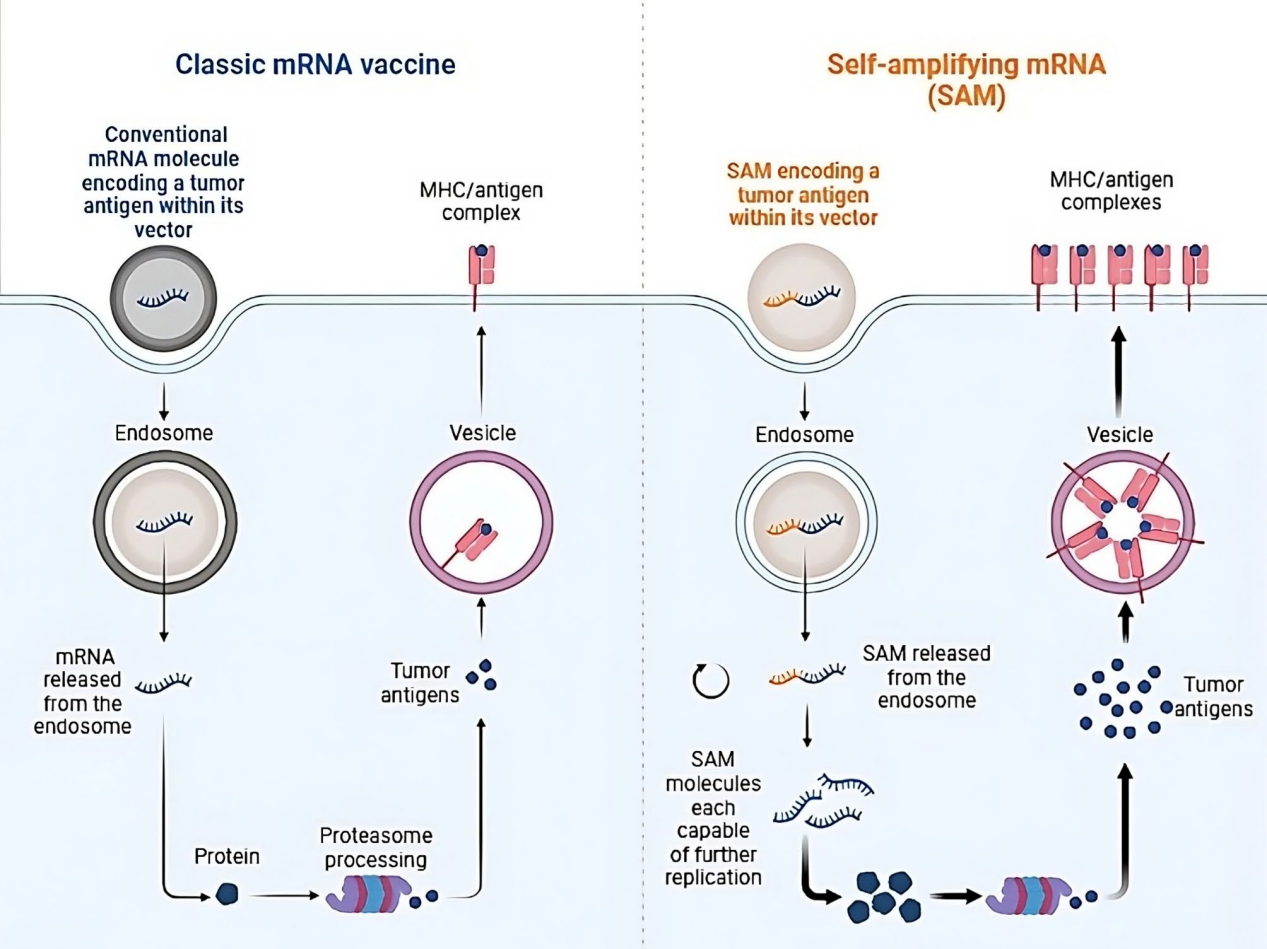
Comparing conventional and self-amplifying mRNA (SAM). Unlike conventional non-replicating mRNA molecules, SAM molecules are capable of self-replication through the incorporation of a sequence in the mRNA molecule which encodes RNA-dependent RNA-polymerase (shown in yellow). This leads to an increase in the production of tumor antigens intracellularly and an increase in its presentation on the cell’s surface which can induce a more potent immune response with a lower dose of the cancer vaccine. Courtesy of Biorender and Maruggi and colleagues [1].

SAM vaccines are derived from positive single-stranded mRNA viruses, most notably alphaviruses including Sindbis virus and Semliki-Forest virus, although negative-sense mRNA viruses have occasionally been employed for SAM design including measles viruses and Rhabdoviruses [24]. SAM molecules are composed of both TA-encoding genes and replicase-encoding genes that code for RNA-dependent RNA-polymerase which acts intracellularly to amplify the genes encoding TAs. In these mRNA molecules, genes encoding viral structural proteins are replaced with genes encoding TAs. The most notable upside of this platform is the enhanced and prolonged antigen production and the decreased required dose. SAM is a huge, negatively charged molecule that is susceptible to various degrading RNases. Also, the internalization process of naked SAM molecules into cells is not efficacious. To overcome these challenges, vectors have been developed to protect the SAM molecules from enzymatic degradation and facilitate the process of internalization [21]. Early clinical applications of SAM used viral replication particles (VRPs) as delivery vehicles posing the risk of toxicity, vector-induced infection, and immunity to viral particles hindering repeated dosing [25, 26]. In order to abolish the risk of infection from viral components, a propagation-defective type of VRP was produced. The envelope and capsid proteins of the modified VRP are encoded in trans as defective helper constructs during production so that just the TA-encoding RNA can be further replicated following internalization [27]. See Fig. 1.

Synthetic vehicles and *D*NA-launched SAM *Rep*licon (DREP) platforms [28] employing plasmids can also be used to deliver the SAM molecules. Medium-length cationic polymer polyethyleneimine (PEI), Pabol, and lipid nanoparticles (LNPs) are just a few examples of synthetic vehicles for SAM delivery [24, 29–31]. These platforms do not induce immunity to vector particles; they are quite safe and pose no infectious risk.

### Trans-amplifying mRNA (taRNA)

taRNA vaccine is a bipartite vector system that has two components, co-transfecting host cells: **A) Vector cassette** (this component is usually derived from the deletion of replicase genes from SAM originating from a virus. This component is sometimes called the trans-replicon (TR)), **B) Replicase component** (this component is provided in trans using several vehicles including uncapped nrRNA produced in IVT, encoding plasmids, or unrelated SAM). TR-replicase proper interaction is ensured by the preservation of other elements on TR such as 3’ and 5’ conserved sequence elements. Compared to SAM, this platform might be more advantageous regarding safety, versatility, and manufacturing [32]. From a versatility point of view, utilizing a taRNA vaccine might be superior to SAM as it allows for independent optimization of the trans-replicon and replicase vector. For instance, the replicase vector can be sequentially optimized without compromising the structural integrity or translation efficiency of the trans-replicon [32]. Moreover, contradictory to the SAM vector that does not tolerate nucleoside modification, taRNA structures can be easily modified to enhance the resulting immune response by augmenting the translation efficiency [33–35]. Usually, the longer the mRNA sequence, the more unstable it is in vivo. Because of this phenomenon, splitting the vector system as in the taRNA, makes the vaccination more cost-efficient compared to SAM as it facilitates the storage and transportation [36]. Also, some experiments have demonstrated that the taRNA vector can elicit the same level of immune response with a considerably lower dose of the vaccine compared to SAM. A group investigated the efficiency of the split-vector system taRNA in immunization against the influenza virus in mice. The vaccine consisted of hemagglutinin-encoding trans-replicons (TR-HA) over a dose range of 0.05–31.25 µg combined with 20 µg of replicase-encoding nrRNA, administered intradermally twice. Interestingly, the lowest doses of TR-HA were associated with HA-specific virus-neutralizing antibody responses comparable to that elicited by human Licenced vaccine (hLIC). The mice survived the influenza virus challenge with no signs of illness [32]. This will further improve the cost-efficiency of the vaccine. Additionally, using taRNA-based vaccines, the process of manufacturing new vaccines can be considerably accelerated which will greatly improve the outcome of cancer patients who are in desperate need of early treatment. To develop a vaccine, the invariable component, which is usually the replicase-encoding vector can be mass-produced and all that is needed would be the insertion of the desired TAs into the trans-replicon. This means using only one bipartite vector system several vaccines targeting many different antigens can be easily produced in a short amount of time [36].

## Advantages and disadvantages of mRNA cancer vaccines

mRNA cancer vaccines have proven to be well-tolerated and safe in clinical and preclinical trials. Unlike conventional methods of cancer treatment, like chemotherapy and radiotherapy, they are far less associated with systemic side effects, producing a systemic immune response that is as effective on metastatic lesions as on the primary tumor [37]. mRNA cancer vaccines can be easily mass-produced in a relatively short time. One of the most important advantages of this vaccine platform is the possibility of flexible design with multiple TAs and the incorporation of immunomodulatory genes (such as Interleukin (IL)-2 and granulocyte-macrophage colony-stimulating factor (GM-CSF)). They also elicit a memory immune response that will lower the incidence of recurrence. Ultimately, human leukocyte antigen (HLA) haplotyping is not required for mRNA vaccine development as it is for peptide-based cancer vaccines [38].

mRNA vaccines also have some advantages over DNA vaccines; because of bypassing the transcription process required for DNA vaccines, mRNA vaccines initiate the expression of the antigen in a shorter time. Furthermore, they don’t pose any genetic risk associated with insertional mutagenesis, as DNA vaccines might. The basic concept of mRNA vaccines has been known for many years but their application has been limited. One of the most important reasons is the intense intrinsic immunogenicity. Specific mRNA motifs and phage RNA-produced dsRNA can activate the innate immune system through pattern recognition receptors (PRRs). In the endosome, toll-like receptor (TLR)-7 and TLR-8 can activate the myeloid differentiation marker 88 pathway upon encounter with mRNAs, whereas in the cytosol mRNA molecules are sensed by other PRR families, such as retinoic acid-inducible gene-I-like (RIG-I-like) receptors, oligoadenylate synthetase (OAS) receptors, and RNA-dependent protein kinase. All these pathways culminate in the secretion of type 1 interferon (IFN) and other pro-inflammatory cytokines. This can paradoxically have a detrimental or beneficial effect on the vaccine’s efficacy. On one hand, by inducing APC activation and maturation, it enhances antigen processing and presentation and boosts the resulting immune response. On the other hand, the activation of downstream signaling pathways upon encounter induces mRNA degradation and mRNA translation blockage through eukaryotic initiation factor 2 (eIF2) phosphorylation and subsequent inactivation [39, 40]. dsRNA species can be reduced during IVT through increasing temperature and decreasing magnesium content [39]. Removal of dsRNA can be achieved through high-pressure liquid chromatography, solid phase synthesis of short mRNA molecules with subsequent ligation of the resulting transcripts, and selective binding of dsRNA to cellulose powder followed by fast protein liquid chromatography [41–44].

## Optimization strategies for efficiency enhancement of mRNA vaccines

**Fig. 2:**
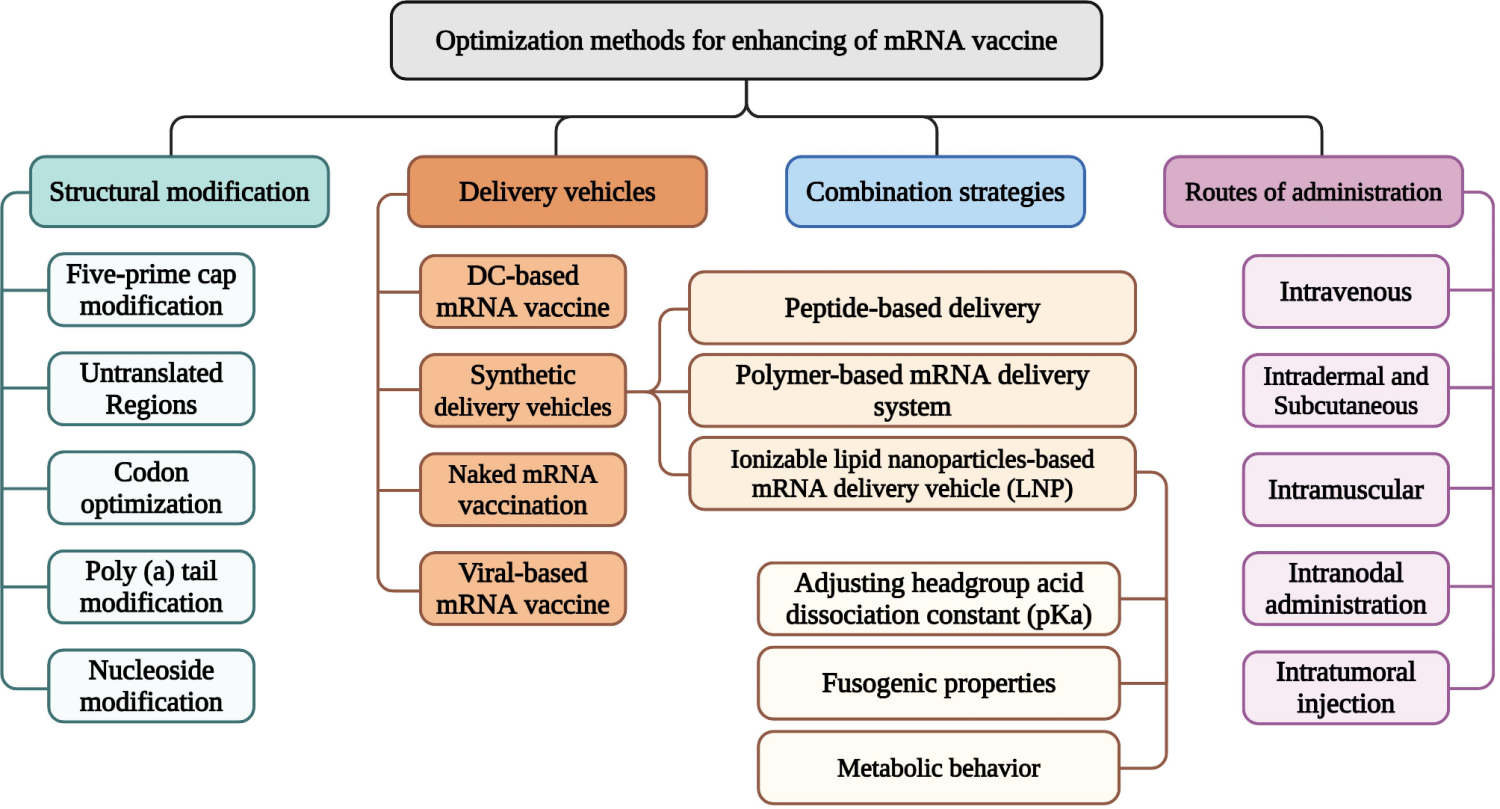
Optimization strategies for mRNA vaccines; these strategies can be classified into four categories. Structural modifications deal with techniques that optimize the structure and sequence of mRNA vaccines to improve their efficiency. Delivery vehicles and routes of administration can be optimized to ensure the efficient transport of antigen-encoding mRNA molecules into APCs. Combination strategies exploit the beneficial therapeutic effects of other anti-cancer therapeutics to improve patient outcomes

To overcome the limitations associated with these vaccines, several strategies have been thought of and implemented (Fig. 2). Some of them will be reviewed in this section.

## Structural modification

### Five-prime cap modification

Mimicking eukaryotic mRNAs, the 5’ ends of IVT-mRNAs possess a 5’-5’ triphosphate linkage. This structure plays important roles in efficient ribosome recognition by eukaryotic initiation factor 4E (eIF4E), mRNA nuclear export, and mRNA stability by blocking degradation by the 5’-3’ exoribonucleases which explains its evolutionary conservation across the eukaryotic domain. This N7 methylated guanosine triphosphate nucleotide is called Cap’0’ [21]. There are two approaches to the incorporation of cap’0’ into the IVT-mRNA, either enzymatically or chemically. The enzymatic approach usually utilizes the Vaccinia capping enzyme (VCE) to incorporate the nucleotide post-transcriptionally, providing a near 100% efficiency [45–47]. Chemical approaches add cap analogs co-transcriptionally which can lead to the reverse incorporation of cap’0’. To overcome this problem, anti-reverse cap analogs (ARCAs) have been developed that are methylated at the C3 position of the ribose moiety, ensuring the addition of the cap in the appropriate orientation. Further modifications of the ARCA aim at inhibiting decapping and increasing eIF4E recognition by incorporation of bridging and non-bridging oxygen [48, 49].

Structures such as cap’1’ and cap’2’ can also be incorporated into the resulting mRNA post-transcriptionally to further improve its stability. Since uncapped mRNA can be recognized by various PRRs including RIG-1 which blocks mRNA translation, IVT-mRNAs must be treated with phosphatases to remove uncapped phosphates to prevent mRNA recognition by these intracellular receptors [50–53].

### Untranslated regions (UTRs)

UTRs can have considerable effects on mRNA stability and translational efficiency [54]. Adding the 3’UTR twice in tandem might help with translation efficiency [55]. Avoiding canonical and non-canonical start codons and highly stable secondary structures in the 5’UTR helps with the normal process of translation and enhances the mRNA translation rate. Studies have demonstrated that shorter 5’UTR sequences are more conducive to mRNA translation [56]. Also, AU- and GU-rich sequences in UTRs can help with RNA stability [55]. Ultimately, the best way to optimize UTR sequences is to understand the biology of targeted cells since UTR performance is dependent on species, cell type, and cell state [21].

### Codon optimization

Studies have demonstrated that codon optimization without chemical alteration of mRNA results in a higher antigen expression rate than the other way around [57]. Several strategies have been proposed to modify codons resulting in a higher TA expression rate, of which some will be discussed here. Higher cytosine and guanine content and lower uridine content are beneficial concerning protein expression rate. Uridine-rich transcripts can be easily recognized by RIG-1, whose activation results in suppressed mRNA translation.

To further improve mRNA translation, one can look into the characteristics of the highly expressed mRNA transcripts of the targeted cell population [58]. For instance, transcripts that have the same ratio of every codon as highly expressed mRNAs in the target cells, usually have higher expression rates. Using codons with more abundant cognate tRNA can augment the translation process as they improve accessibility to amino acids during translation, although the accuracy of this method has been questioned [59]. As mentioned before, highly stable secondary structures and hairpin loops should be avoided, as they interfere with the normal translation process [39]. However, a high expression rate has its downsides as it can interfere with the folding and post-translational modification of some proteins. Therefore, the expression rate shall be monitored intently to ensure translational accuracy [21].

### Poly (A) tail modification

Poly (A) tail can help prevent mRNA degradation through RNase activity and help with translation efficiency. This segment of the mRNA can be added to the structure using poly-a polymerases or be encoded in the original transcript [60]. The optimal length of the poly (A) tail for monocyte-derived DCs (MoDCs) is 120–150 nucleotides [39]. Poly (A) binding proteins (PABP) are known to interact with the 5’ cap through eIF4g and eIF4E, forming a closed loop that can enhance translation [39, 61]. Lima et al. demonstrated that shorter poly (A) sequences are more suitable for the formation of these closed loops [61].

### Nucleoside modification

Intracellular anti-viral immunity mechanisms can recognize an mRNA transcript based on its nucleosides. These mechanisms can lead to the activation of pattern recognition receptors and double-stranded RNA-dependent protein kinase among others, which will suppress the translation of mRNA and promote its degradation [62]. To overcome this issue, one strategy is to use nucleoside-modified mRNA molecules. The groundbreaking works of Karikó and colleagues showed that the incorporation of modified nucleosides such as 5-methylcytidine (m5C), 6-methyladenine (m6A), 5-methyluridine (m5U), and 2-thiouridine (s2U) into the structure of mRNA can lead to the ablation of anti-mRNA responses [63]. Also, Kormann and colleagues demonstrated that modified mRNA nucleosides such as 5-methylcytidine can prevent RNA-induced immune responses and promote mRNA expression [64].

The incorporation of pseudouridine (Ψ), 1-methyl pseudouridine (m1Ψ), and m5C has been associated with enhanced antigen expression which is relatable to the removal of RIG-1 danger signals associated with the usage of cytidine and uridine [41, 65–67]. Post-transcriptional modification of RNA with N4-acetylcytidine showed enhanced mRNA translation both in vivo and in vitro [68]. Also, a methyl-transferase called mettl3, which works by 6-methylation of adenine shows promising results in DC activation assays [39, 69].

## Delivery vehicles

**Fig. 3:**
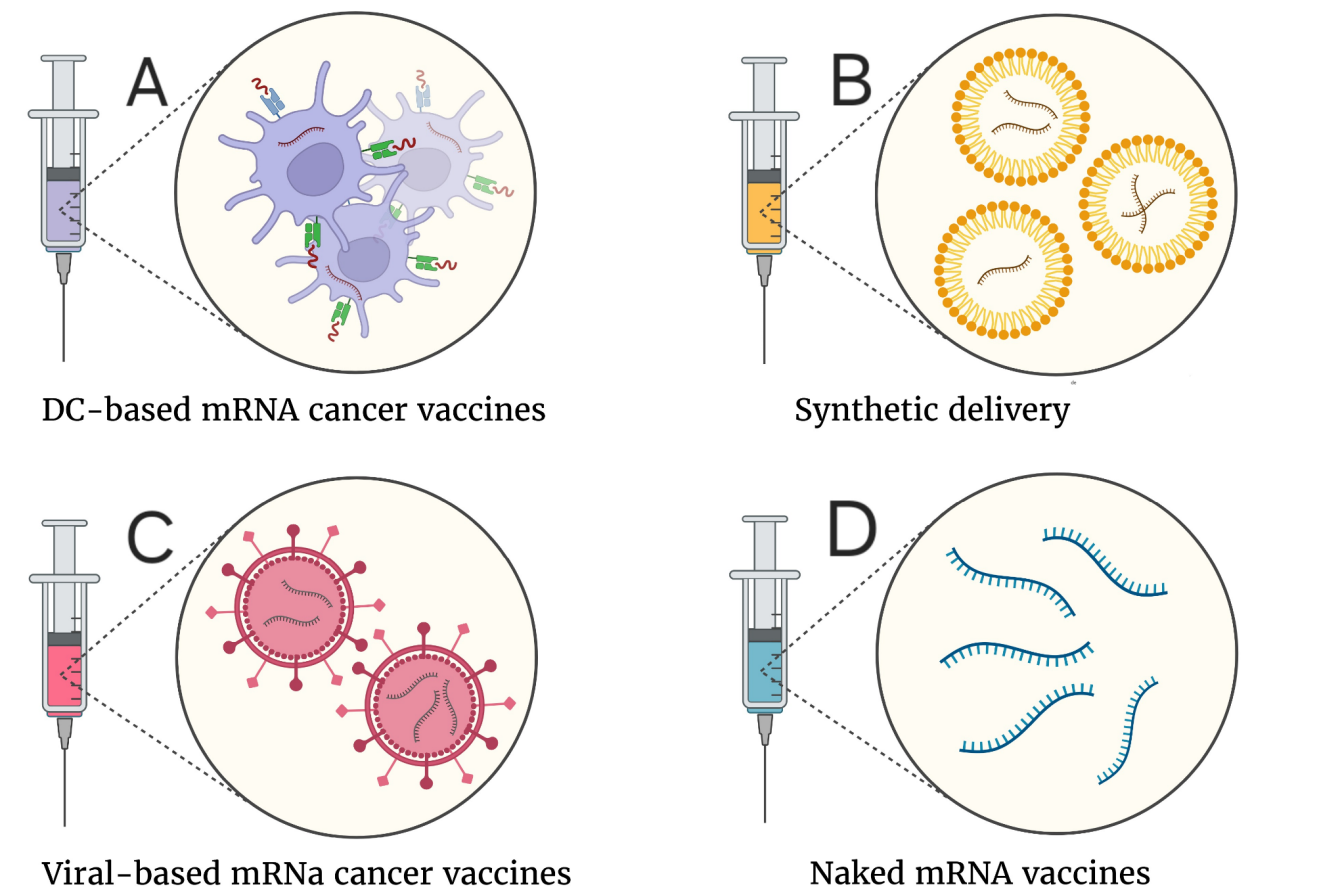
Different platforms of mRNA vaccination delivery (**A**) In DC-based mRNA cancer vaccines, the peripheral blood mononuclear cells (PBMCs) are extracted from the patient’s blood, treated with several maturation factors and loaded with TA-encoding mRNA to be reinfused into the patient (**B**) In synthetic delivery methods, the mRNA is encapsulated in a synthetic vector (such as a liposome as demonstrated in the figure) to be injected into the patient (**C**) In a virus-based mRNA cancer vaccine, the antigen-encoding MRNA is encapsulated in a virus or virus-like particle to be infused into the patient (**D**) In naked mRNA vaccination, the mRNA molecule is delivered into the patient with any conjugates. Because of this, it is vulnerable to various nucleases in the body following administration

A hot topic in the field of cancer research is optimizing the delivery vehicles for optimal transfection of APCs and subsequent T-cell-mediated immunity [70–73]. Here, some of the currently applied delivery vectors for the mRNA cancer vaccines are discussed (Fig. 3).

### DC-based mRNA cancer vaccines

**Fig. 4:**
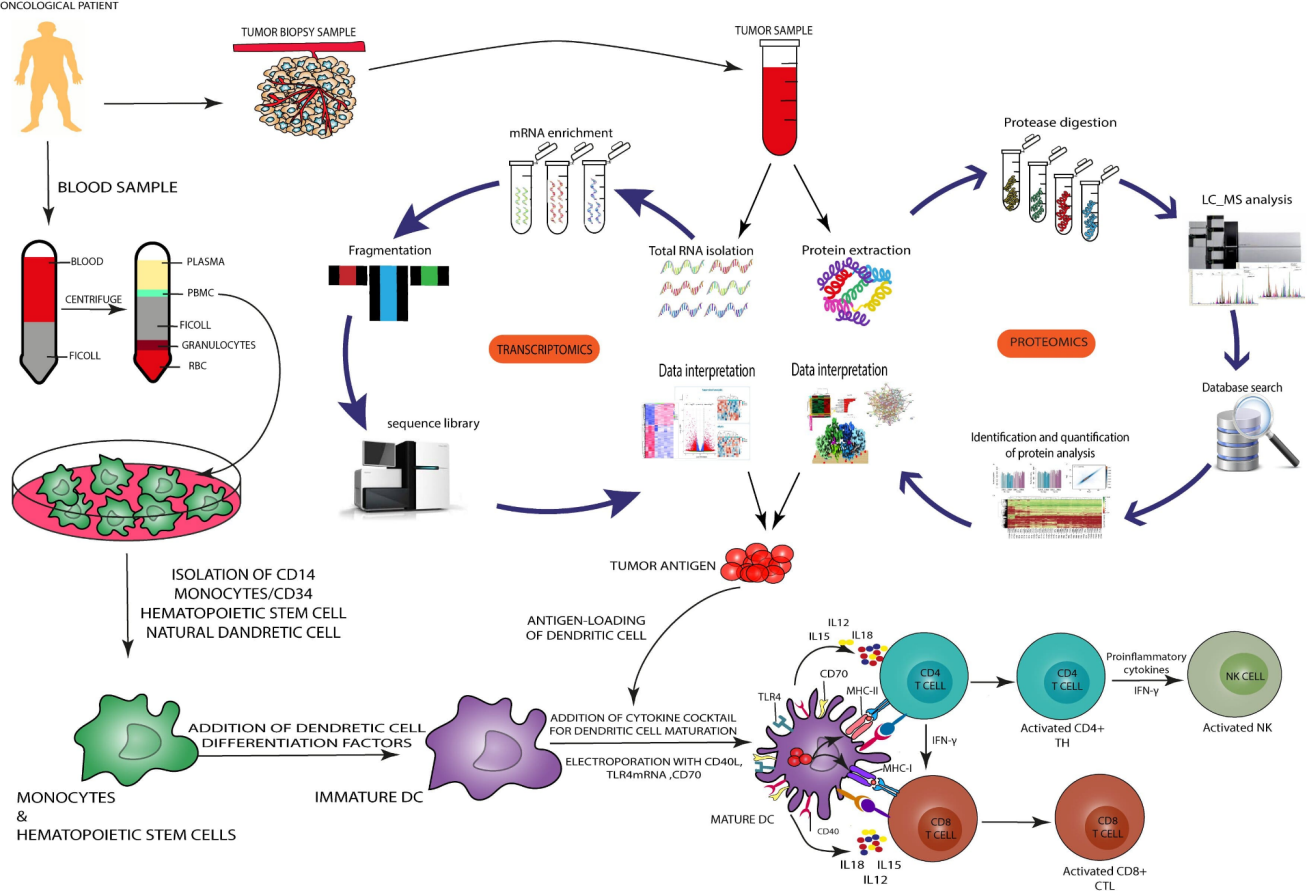
Induction of anti-tumor immune response by DC. In order to generate DCs, first isolate the PBMC cells using the Ficoll gradient, and then purify the monocytes (CD14+), hematopoietic stem cells (CD34+), and DCs. Then, using differentiating factors, these cells are differentiated into immature DCs. To produce TAs, the cancer specimen is analyzed using transcriptomics and proteomics methods. By using bioinformatics analysis of the resulting data, it is possible to identify the protein structure and gene sequence of the desired antigen. After the transfer of the desired highly immunogenic antigens and their uptake by the DCs, through the cytokine cocktail and the transfection of gene sequences CD40L, CD70, and TLR4, stimulation signals to transform the immature DCs into mature cells are induced. Returning these mature cells to the patient efficiently leads to the activation of CD4 + and CD8 + cells through antigen presentation, which subsequently activates the NK and B cells. Finally, an effective immune response is created against TAs

Autologous ex vivo-loaded DCs are the perfect vector for mRNA cancer vaccination as DCs are the most well-equipped APCs. This platform allows for the precise targeting of DCs with high transfection efficiency [74]. DCs are optimally amenable to mRNA transfection through various endocytic processes and electroporation. The production is divided into two converging pathways. For the vector acquisition step, a blood sample is drawn from the oncological patient to be used for autologous peripheral blood mononuclear cells (PBMC) extraction. These PBMCs are treated with differentiation factors to obtain immature DCs. Then, these immature DCs are treated with a cocktail of cytokines to produce mature antigen-presenting DCs. For the antigen acquisition step, a tumor biopsy is usually indicated. One can detect antigens in the biopsy specimen through proteomic, transcriptomic, and genomic analysis. For genomic analysis, different DNA sequencing methods are available, out of which the relatively new next-generation sequencing (NGS) has sparked a revolution because it requires a much shorter time to do the job. For proteomic analysis, firstly, the proteins are extracted and treated with proteases. The resulting peptides can go through different peptide sequencing procedures including a liquid chromatography-mass spectrometry analysis apparatus. By comparing these sequences with those of a database, one can identify the TAs. For a transcriptomic analysis, after mRNA extraction, enrichment, and fragmentation, the mRNA sequence is determined which can be used to identify TAs. Following a screening process for the identification of the most immunogenic antigens, the desired TAs are encoded in an mRNA molecule. At this point, the two pathways converge as the mRNA molecules are transfected into DCs. These mRNA-loaded DCs can be delivered into the patient’s body through a plethora of routes. Once inside the body, the expressed TA is presented on MHC molecules to induce a therapeutic and protective anti-tumor immune response (Fig. 4) [75, 76].

The first proof-of-concept study of an mRNA-electroporated DC vaccine was conducted by Boczkowski et al. This experiment demonstrated the induction of a robust immune response following the transfection of DCs with ovalbumin (OVA) [77]. DC-loaded mRNA vaccines are primarily administered via intradermal and intravenous routes, but there have been instances of intranodal administration as well (NCT01278940, NCT01530698). See Table 1.

**Table 1:** Clinical trials that include mRNA cancer vaccines into their treatment regimen

NCT	Phase	Status	Indication	Formulation type	Route	Sponsor
NCT01066390	Phase I	Completed	Stage III/IV malignant melanoma (Unresectable)	Dendritic cells expressing TriMix, MAGE-A3, MAGE-C2, tyrosinase, gp100	IV and ID	Universitair Ziekenhuis Brussel
NCT01676779	Phase II	Completed	Melanoma	Dendritic cells expressing TriMix, MAGE-A3, MAGE-C2, tyrosinase, gp100	IV and ID	Universitair Ziekenhuis Brussel
NCT01302496	Phase II	Completed	Stage III/IV Malignant Melanoma (Unresectable)	Dendritic cells expressing TriMix, MAGE-A3, MAGE-C2, tyrosinase, gp100 + IV ipilimumab	IV and ID	Bart Neyns/ Vrije UniversiteitBrussel
NCT01278940	Phase I/II	Completed	Melanoma	DCs loaded with autologous tumor or TAA-encoding mRNA + IL-2	ID and IN	Oslo University Hospital
NCT00678119	Phase II	Completed	Renal cell carcinoma	DCs loaded with total tumor mRNA and CD40L mRNA + sunitinib	ID	Argos Therapeutics
NCT00510133	Phase II	Completed	AML (complete remission)	DCs loaded with hTERT mRNA containing a LAMP-1 targeting sequence	ID	Asterias Biotherapeutics, Inc.
NCT01446731	Phase II	Completed	Metastatic prostate cancer	DCs loaded with mRNA encoding PSA, PAP, survivin, hTERT + Docetaxel	ID	Herlev Hospital
NCT02285413	Phase II	Completed	Melanoma	DCs loaded with mRNA encoding tyrosinase, gp100 + cisplatin	ID and IV	Radboud University
NCT00626483	Phase 1	Completed	Malignant neoplasms of the brain	DCs loaded with mRNA encoding CMV pp65-LAMP + basiliximab	-	Duke University
NCT01278914	Phase I/II	Completed	Androgen-resistant metastatic prostate cancer	DCs loaded with tumor-associated antigen mRNA	-	Oslo University Hospital
NCT02529072	Phase I	Completed	Malignant Glioma, Astrocytoma, Glioblastoma	DCs loaded with CMV-pp65 encoding mRNA + nivolumab and surgical resection	-	Duke university
NCT00890032	Phase I	Completed	Recurrent Central Nervous System Neoplasm	DCs loaded with brain tumor stem cell (BTSC) encoding mRNA	ID	Duke university
NCT00846456	Phase I/II	Completed	Glioblastoma	DCs loaded with brain tumor stem cell (BTSC) encoding mRNA	ID	Oslo University Hospital
NCT01530698	Phase I/II	Completed	Melanoma	DCs loaded with mRNA encoding gp100, tyrosinase, active TLR4, and CD70	IN	Radboud University
NCT00940004	Phase I/II	Completed	Melanoma	DCs loaded with mRNA encoding gp100, tyrosinase, and either cytokines or TLR ligands	ID and IV	Radboud University
NCT00243529	Phase I/II	Completed	Melanoma	DCs loaded with mRNA encoding gp100 and tyrosinase	-	Radboud University
NCT00228189	Phase I/II	Completed	Colorectal cancer	DCs loaded with mRNA encoding CEA	ID and IV	Radboud University
NCT00978913	Phase I	Completed	Breast cancer	DCs loaded with mRNA encoding hTERT, survivin, and p53 if the tumor expresses p53 + cyclophosphamide	ID	Herlev Hospital
NCT01734304	Phase I/II	Completed	AML	DCs loaded with mRNA encoding WT1, PRAME, and CMVpp65	ID	Ludwig-Maximilian-University of Munich
NCT00834002	Phase I	Completed	AML	DCs loaded with mRNA encoding WT1	ID	Antwerp University Hospital
NCT00087984	Phase I/II	Completed	RCC	DCs loaded with autologous tumor mRNA with or without CD40L mRNA	-	Argos Therapeutics
NCT00272649	Phase I/II	Completed	RCC	DCs loaded with autologous tumor mRNA with or without CD40L mRNA	-	Argos Therapeutics
NCT00639639	Phase I	Completed	Glioblastoma	DCs loaded with mRNA encoding CMV pp65-LAMP with or without autologous lymphocyte transfer	ID	Duke University
NCT02366728	Phase II	Completed	Glioblastoma	DCs loaded with mRNA encoding CMV pp65-LAMP + Basiliximab and Temozolomide	ID	Duke University
NCT03615404	Phase I	Completed	Glioblastoma	DCs loaded with mRNA encoding CMV pp65-LAMP with or without autologous lymphocyte transfer + GM-CSF and Temozolomide	ID	Duke university
NCT02808416	Phase I	Completed	Brain metastases	DCs loaded with mRNA encoding patient-specific TAAs	ID and IV	Guangdong 999 Brain Hospital
NCT02808364	Phase I	Completed	Recurrent glioblastoma	DCs loaded with mRNA encoding patient-specific TAAs	ID and IV	Guangdong 999 Brain Hospital
NCT02709616	Phase I	Completed	Glioblastoma	DCs loaded with mRNA encoding patient-specific TAAs + Temozolomide	ID and IV	Guangdong 999 Brain Hospital
NCT01456104	Phase I	Completed	Melanoma	Langerhans-type DCs loaded with Trp2 mRNA	-	Memorial Sloan Kettering Cancer Center
NCT01995708	Phase I	Completed	Multiple myeloma	Langerhans-type DCs loaded with CT7, MAGE-A3, WT1 mRNA	ID	Memorial Sloan Kettering Cancer Center
NCT01686334	Phase II	Recruiting	AML	DCs loaded with WT1 mRNA + low-dose chemotherapy	ID	Antwerp University Hospital
NCT02649582	Phase I/II	Recruiting	Glioblastoma	DCs loaded with WT1 mRNA + temozolomide and temozolomide-based chemoradiation	ID	Antwerp University Hospital
NCT02649829	Phase I/II	Active	Malignant pleural mesothelioma	DCs loaded with WT1 mRNA + conventional chemotherapy	ID	Antwerp University Hospital
NCT04157127	Phase I	Recruiting	Pancreatic cancer	DCs loaded with tumor-cell lysate Adjuvant to chemotherapy	ID	Baylor College of Medicine
NCT03688178	Phase II	Active	Glioblastoma	DCs loaded with CMV pp65-LAMP mRNA + Temozolomide and varlilumab	ID	Duke University
NCT04335890	Phase I	Active	Uveal metastatic melanoma	DCs loaded with mRNA encoding TAAs (gp100, tyrosinase, PRAME, MAGE-A3, IDO) and TSAs (GNAQ/GNA11Q209 or R183)	IV	Hasumi International Research Foundation
NCT02465268	Phase II	Active	Glioblastoma, malignant glioma, astrocytoma	DCs loaded with mRNA encoding CMV pp65-LAMP Combined with GM-CSF	ID	Immunomic Therapeutics, Inc.
NCT01983748	Phase III	Active	Uveal melanoma	DCs loaded with autologous tumor mRNA	IV	University Hospital Erlangen
NCT03396575	Phase I	Recruiting	Diffuse intrinsic pontine glioma, Brain stem glioma	DCs loaded with total tumor mRNA + GM-CSF, Cyclophosphamide, Fludarabine, and intravenous infusion of ex vivo expanded tumor-reactive lymphocytes	ID	University of Florida
NCT01326104	Phase I/II	Active	Medulloblastoma, Neuroectodermal tumor	DCs loaded with total tumor mRNA + ex vivo expanded tumor-reactive lymphocytes	ID and IV	University of Florida
NCT01197625	Phase I/II	Active	Prostate cancer	DCs loaded with mRNA From Primary Prostate Cancer Tissue, also encoding hTERT, and Survivin	-	Oslo University Hospital
NCT05000801	Not applicable	Recruiting	AML	DCs loaded with mRNA encoding WT1, hTERT, and survivin	-	Affiliated Hospital to Academy of Military Medical Sciences
NCT05799612	Phase I	Not yet recruiting	Cutaneous angiosarcoma	DCs loaded with tumor mRNA and tumor lysate + paclitaxel, pegylated-IFN α, and filgrastim (a recombinant form of G-CSF)	IV	M.D. Anderson Cancer Center
NCT04837547	Phase I	Recruiting	Neuroblastoma, Diffuse intrinsic pontine glioma	DCs loaded with total tumor mRNA + tumor-specific ex vivo expanded autologous lymphocyte transfer and autologous G-CSF mobilized hematopoietic stem cells	-	Wake Forest University Health Sciences, University of Florida
NCT04911621	Phase I/II	Active	High-grade glioma, Diffuse intrinsic pontine glioma	DCs loaded with WT1-encoding mRNA + chemoradiotherapy	ID	University Hospital, Antwerp
NCT00204516	Phase I/II	Completed	Melanoma	Naked mRNA TAA for melanoma (Melan-A, Mage-A1, Mage-A3, survivin, GP100, and tyrosinase) + GM-CSF	ID	The Norwegian Radium Hospital
NCT02035956	Phase I	Completed	Melanoma	Naked mRNA, Neo-Ag/ TAA (IVAC MUTANOME, RBL001/RBL002)	Ultrasound-guided IN	BioNTech
NCT01684241	Phase I	Completed	Melanoma	Naked tumor-associated antigen or neo − Ag mRNA (RBL001/RBL002)	IN	Pharmaceuticals GmbH
NCT03788083	Phase I	Recruiting	Early-stage Breast Cancer	Naked Trimix mRNA (mRNA encoding CD40L, CD70, acTLR4)	Intratumoral	Universitair ZiekenhuisBrusse, eTheRNA immunotherapies
NCT00923312	Phase I/II	Completed	Stage IIIB/IV NSCLC	RNActive, (Protamine) MAGE-C1, MAGEC2, NY-SEO-1, survivin, 5 T4	ID	CureVac
NCT03164772	Phase I/II	Completed	NSCLC	RNActive (Protamine) NY-ESO-1, MAGEC1, MAGE-C2, 5 T4, survivin, MUC1 + Durvalumab, Tremelimumab	ID	CureVac
NCT00831467	Phase I/II	Completed	Prostate cancer	RNActive (Protamine) encoding PSA, PSCA, PSMA, STEAP1, PAP, MUC1	ID	CureVac
NCT00204607	Phase I/II	Completed	Melanoma	Protamine-complexed tumor-associated antigen mRNA encoding Melan-A, Mage-A1, Mage-A3, Survivin, GP100 and Tyrosinase + GM-CSF	ID	University Hospital Tübingen
NCT02410733	Phase I	Completed	Melanoma	Lipo-MERIT, DOTMA (DOTAP)/ DOPE lipoplex encoding NY-ESO-1, MAGEC3, tyrosinase, gp100	IV	BioNTech
NCT02316457	Phase I	Completed	Triple-negative breast cancer (TNBC)	Lipo-MERTI, DOTMA(DOTAP)/ DOPE lipoplex	IV	BioNTech
NCT05264974	Phase I	Not yet recruiting	Melanoma (subjects who progressed on anti-PD1 therapy)	Autologous total tumor mRNA loaded DOTAP liposome vaccine	IV	University of Florida
NCT03948763	Phase I	Completed	Colorectal cancer, NSCLC, Pancreatic cancer	LNP KRAS mutations: G12D, G12V, G13D, G12C + Pembrolizumab	IM	Moderna, Merck
NCT03739931	Phase I	Recruiting	Dose Escalation: Relapsed/Refractory Solid Tumor Malignancies or Lymphoma Dose Expansion: Other solid tumors	LNP mRNA-2752 (mRNA encoding OX40L, IL-23, IL- 36Ƴ), alone (Phase I) or + i.v. PD-L1 inhibitor, Durvalumab (Durva, Phase II)	Intratumoral	ModernaTX, Inc., AstraZeneca
NCT03289962	Phase I	Active	Melanoma, NSCLC, Bladder Cancer, CRC, Breast Cancer	Lipo-MERIT Neo-Ag (mRNA) + atezolizumab	IV	BioNTech, Genentech
NCT03815058	Phase II	Active	Advanced Melanoma	Lipo-MERIT Neo-Ag (mRNA) + pembrolizumab	IV	BioNTech, Genentech
NCT04486378	Phase II	Recruiting	Stage II and III CRC (surgically resected)	Lipo-MERIT Neo-Ag (mRNA)	IV	BioNTech
NCT04161755	Phase I	Active	Pancreatic Cancer (surgically resected)	Lipo-MERIT Neo-Ag (mRNA) + Atezolizumab and FOLFIRINOX	IV	Memorial Sloan Kettering Cancer Center, Genentech
NCT03313778	Phase I	Active	Mono-: resected solid tumors; Combo: unresectable solid tumor	LNP Neo-Ag (mRNA) + pembrolizumab	IM	Moderna, Merck
NCT03897881	Phase II	Recruiting	Complete resection of High-Risk Melanoma	LNP Neo-Ag (mRNA) + pembrolizumab	IM	Moderna, Merck
NCT05660408	Phase I/II	Not yet recruiting	Recurrent pulmonary osteosarcoma	LNP loaded with TA-encoding mRNA	IV	University of Florida
NCT04573140	Phase I	Recruiting	Adult glioblastoma	Autologous total tumor mRNA and pp65- LAMP mRNA loaded DOTAP liposome vaccine	IV	University of Florida
NCT04382898	Phase I/II	Recruiting	Prostate cancer	RNA-LPX encoding five prostate TAAs (BNT112) + cemiplimab	IV	BioNTech SE
NCT04534205	Phase II	Recruiting	HPV16 + and PD-L1 + Head and neck squamous cell carcinomas (HNSCC)	RNA-LPX encoding HPV16 antigens + pembrolizumab	IV	BioNTech SE
NCT04526899	Phase II	Recruiting	Melanoma	RNA-LPX encoding NY-ESO-1, MAGE-A3, tyrosinase and TPTE + Cemiplimab	IV	BioNTech SE
NCT04503278	Phase I/II	Recruiting	Solid tumors	RNA-LPX encoding CLDN6 + CLDN6-specific CAR-T cells	IV	BioNTech SE
NCT01890213	Phase I	Completed	Stage III colorectal cancer	SAM Alphavirus replicon (VRP) encoding CEA	IM	AlphaVax
NCT00529984	Phase I/II	Completed	Advanced or metastatic CEA-expressing solid tumor	SAM Alphavirus replicon (VRP) encoding CEA	IM	AlphaVax
NCT05916248	Phase I	Recruiting	Advanced solid tumors	mRNA cancer vaccine encoding tumor neo-antigens with or without pembrolizumab	-	Ruijin Hospital, Shanghai XinpuBioTechnology Company Limited
NCT05714748	Phase I	Recruiting	EBV-related malignancies	mRNA vaccine encoding EBV antigens	IM	West China Hospital
NCT03908671	Not applicable	Recruiting	Esophageal Cancer, Non-Small Cell Lung Cancer	mRNA vaccine encoding neo-antigens	SC	Stemirna Therapeutics
NCT05192460	Not applicable	Recruiting	Gastric Cancer, Esophageal Cancer, Liver Cancer	mRNA vaccine encoding neo-antigens with or without anti-PD-1/PD-L1	ID	jianmingxu, NeoCura
NCT05942378	Phase I	Not yet recruiting	Advanced solid tumors	mRNA cancer vaccine (HRXG-K-1939) + adebrelimab	-	Fudan University
NCT05949775	Not applicable	Not yet recruiting	Advanced solid tumors	mRNA vaccine encoding neo-antigens + sintilimab	SC	Stemirna Therapeutics
NCT05738447	Phase I	Recruiting	Hepatocellular carcinoma	mRNA vaccine encoding HBV antigens	IM	West China Hospital
NCT05198752	Phase I	Recruiting	Advanced solid tumors	mRNA cancer vaccine encoding neo-antigens (SW1115C3)	SC	Stemirna Therapeutics
NCT05761717	Not applicable	Not yet recruiting	Hepatocellular carcinoma (Post-operation)	mRNA cancer vaccine encoding neo-antigens + sintilimab	SC	Shanghai Zhongshan Hospital
NCT05940181	Not applicable	Not yet recruiting	Advanced solid tumors	mRNA cancer vaccine encoding neo-antigens (XH001) + sintilimab	ID	jianmingxu, NeoCura
NCT05916261	Phase I	Recruiting	Advanced pancreatic cancer	mRNA cancer vaccine encoding neo-antigens (mRNA-0217/S001) + pembrolizumab	-	Ruijin Hospital
NCT05938387	Phase I	Recruiting	“MGMT-unmethylated” Glioblastoma	CV09050101 mRNA vaccine (CVGBM)	IM	CureVac
NCT05981066	Not applicable	Recruiting	Advanced hepatocellular carcinoma	mRNA cancer vaccine encoding neo-antigens (ABOR2014/IPM511)	IM	Peking Union Medical College Hospital
NCT05227378	Not applicable	Not yet recruiting	Gastric cancer	mRNA cancer vaccine encoding neo-antigens with or without anti-PD-1/PD-L1	ID	Shen Lin, NeoCura
NCT05579275	Phase I	Recruiting	Advanced solid tumors	mRNA cancer vaccine encoding neo-antigens (JCXH-212)	-	Peking University Cancer Hospital & Institute

Several studies have shown that combining these vaccines with co-stimulatory molecules such as CD83, OX40, CD137L, or IL-12 results in a more potent immune response, as these vaccines are not strong enough to be used as monotherapies [78–81]. TriMix, an mRNA molecule encoding for CD40L, CD70, and constitutively active TLR-4 (caTLR4), can be co-transfected with another mRNA encoding the actual TA(s) enhancing the resulting immune response. However, it should be clear that these immunomodulator-encoding mRNA molecules are not classified as mRNA vaccines. mRNA vaccines encode tumor-associated antigens (TAAs) or tumor-specific antigens (TSAs). The vaccination of stage III and stage IV melanoma patients with DCs loaded with mRNA encoding TAAs plus TriMix adjuvant led to tumor regression in 27% of patients [82].

Transfecting DCs with several TAs or even whole tumor-cell-derived RNA and co-administration of ICB therapies are also of interest, as they can cause broader and more effective immune responses. AGS-003 (Rocapuldencel-T), an autologous MoDC vaccine, loaded with the patient’s tumor-derived RNA in combination with CD40-ligand RNA, co-administered with sunitinib, an ICB, showed promising results in a phase 2 clinical trial in subjects newly diagnosed with advanced kidney cancer [83, 84]. Multiple myeloma (MM) is a common hematologic malignancy. With high-dose chemotherapy followed by autologous stem cell transplantation (ASCT), progression-free survival (PFS) and overall survival (OS) are improved, but virtually every patient will eventually relapse [85–87]. Vaccination against TAs might be a viable option to destroy the remaining tumor cells following chemotherapy and ASCT. In the phase I clinical trial, autologous Langerhans-type DCs were electroporated with mRNA molecules encoding Cancer testis antigen 7 (CT7), Melanoma-associated antigen (MAGE) A3, and Wilms tumor 1 (WT1) and administered intradermally into patients with multiple myeloma following ASCT. The vaccination group received lenalidomide 3 months following transplantation which has been demonstrated to be efficacious as a maintenance therapy following ASCT. The control group did not receive the mRNA-DC vaccination but received lenalidomide after ASCT with the same schedule as the vaccination group. No significant vaccine-related adverse events were reported except for some mild delayed-type hypersensitivity. Immunological responses regarding CD4 + and CD8 + T-cells were increased compared to the control group as were the pro-inflammatory cytokine secretion such as IFN-γ, IL-2, and tumor necrosis factor (TNF)-α. CD107a which is a degranulation marker was also upregulated in the vaccination group. Although the study was not designed to evaluate the clinical efficacy, partly because of the limited number of subjects, the vaccination group showed a mildly more favorable clinical outcome. All in all, this trial demonstrated the safety and immunogenicity of the mRNA-DC vaccine in patients with MM following ASCT [88].

### Naked mRNA vaccination

In vivo delivery of naked unmodified mRNA preferentially transfecting APCs as in intradermal and intranodal injections has been reported to be successful [89–94]. Lymph nodes are immunocompetent tissues scattered in the body containing a plethora of immune cells such as APCs (macrophages and DCs) and lymphocytes (T-cells and B-cells). This makes them an ideal site for vaccination. Typically the needle is inserted into the border of the cortex and medulla of lymph nodes under ultrasonography guidance prior to injection [95]. Notably, a report showed that periodic intranodal immunizations with naked, unmodified mRNA encoding tumor-associated neoantigens can generate robust T-cell immune responses [93, 96]. Although this platform is swift and cost-effective, it does not allow for efficient cell-type-specific delivery [74].

Intranodal administration of naked mRNA is an unconventional but efficacious platform for cancer vaccination. This method allows for targeted antigen delivery to APCs at the site of T-cell activation, precluding the need for APC migration to lymph nodes [74]. Intrasplenic administration has induced the same responses [97]. Co-administration with TriMix or DC-activating proteins such as Flt3L has been shown to further activate a strong immune response [98–100].

Intratumoral mRNA vaccination offers the advantage of rapid and specific activation of tumor-resident T-cells. The introduced antigens can be tumor-related. Also, non-tumor-related transcripts aim at overcoming the hostile immune-inhibitory tumor environment through non-specific activation of the immune system. A naked mRNA coding for GLB-1 provided protection in a glioblastoma mouse model, taking advantage of the immunogenic properties of mRNA [101]. Other formulations including TriMix or mRNAs encoding immune-stimulatory cytokines have also been studied with enhanced immune responses [102, 103].

These vaccines can also be administered in intramuscular [104], intradermal [35], and subcutaneous [105] routes.

### Synthetic delivery vehicles

This category consists of numerous man-made nanoparticle delivery vehicles, providing a synthetic means of mRNA delivery. Nano-medicine is the science of applying nano-materials for the purpose of diagnosing, treating, or preventing health disorders [106]. It has been widely studied and used since its conception in the 1990s. Novel therapeutics often utilize nano-medicine because of its greater therapeutic efficacy compared to other therapeutics and its potential to enhance bioavailability by surface-functionalization [106].

The major categories are as follows: **Ionizable lipid nanoparticles-based mRNA delivery vehicle**, **Polymer-based mRNA delivery systems**, **and Peptide-based mRNA delivery**.

#### Ionizable lipid nanoparticles-based mRNA delivery vehicle

LNP was originally designed to deliver small interfering RNAs (siRNAs) to silence the desired genes through complementary interaction with encoding mRNAs, interfering with the translation. It has proven to be one of the most clinically translatable non-viral mRNA delivery vehicles [107–110]. It is composed of an amino lipid, helper phospholipid, cholesterol, and lipid-anchored polyethyleneglycol (PEG).

The ionizable lipid acquires positive charges by protonation in environments with low pH [111]. This is useful for two reasons: First, this process facilitates encapsulation through interaction with the negatively-charged mRNA during the production phase; secondly, this positive charge can help with the interaction of the liposome and the endosomal membrane upon encounter with the low pH of the endosomal microenvironment, mediating endosomal escape.

The amino group remains neutral in the physiologic pH of the body fluids, increasing stability and decreasing toxicity [21]. In order to further reduce cytotoxicity, biodegradable LNP constituents have been developed. The lipid-anchored PEG prevents macrophage-mediate clearance and aggregation of LNPs, helping with efficiency and stability [112].

LNPs have been designed to protect mRNAs from extracellular RNases and to deliver mRNAs specifically to APCs [21]. LNP-loaded mRNA cancer vaccines are primarily administered intravenously, but there have been instances of intramuscular (NCT03948763) and intratumoral (NCT03739931) delivery as well. See Table 1.

In a phase I clinical trial, Autogene cevumeran, a mRNA-lipoplex vaccine encoding tumor neoantigens was injected intravenously as adjuvant therapy following surgical resection of pancreatic ductal adenocarcinoma together with atezolizumab (an anti-PD-L1 monoclonal antibody) and mFOLFIRINOX chemotherapy regimen. Neoantigen-specific T-cell response was observed in 8/16 patients (known as responders) with 4 patients having responses against more than one neoantigen. Immune responders had a significantly longer recurrence-free survival compared to non-responders [113].

In a phase IIb clinical trial conducted by Moderna and Merck & Co, a personalized neoantigen-based mRNA vaccine (mRNA-4157) was assessed in patients with completely resected high-risk cutaneous melanoma. One group received mRNA-4157 plus pembrolizumab while the other group received pembrolizumab monotherapy. Pembrolizumab is an anti-PD1 antibody that can promote an immune response against tumors, especially if the tumor has a high mutation burden. The vaccine was administered intramuscularly. It was demonstrated that recurrence-free survival was longer with combination compared to monotherapy. No vaccine-related grade 4 or 5 adverse events were observed while 25% of patients in the combination group experienced grade 3 adverse events compared to 18% in the monotherapy group [114].

In a similar phase I trial, patients with resected non-small cell lung cancer (NSCLC) or resected cutaneous melanoma received mRNA-4157 as a monotherapy or combined with pembrolizumab. Using IFN-γ enzyme-linked immunosorbent spot (ELISpot), neoantigen-specific CD4 + and CD8 + T-cell responses were monitored. It was determined that combination therapy drives a more potent and expansive T-cell response against targeted neoantigens with more cytotoxic potential. (NCT03313778)

In another phase I/II trial sponsored by the University of Florida, tumor mRNA-loaded LNPs are administered intravenously into patients with recurrent pulmonary osteosarcoma as research has shown that LNPs loaded with mRNA primarily accumulate in lungs, a fact which could be exploited to treat pulmonary malignancies. The primary objective is to determine the maximum tolerated dose and 12-month recurrence-free survival. To our knowledge, no results of the trial have been released yet. (NCT05660408)

Optimization of LNP-delivered mRNA vaccines relies on **adjusting headgroup acid dissociation constant (pKa)**, **fusogenic properties**, and **metabolic behavio**r.

##### Adjusting headgroup acid dissociation constant (pKa)

This modification can have a tremendous effect on in vivo antigen expression and immunogenicity. The proposed headgroup pKa for the intravenous route of administration is 6.2–6.5, while Hassett et al. reported 6.6–6.9 to be the perfect range for intramuscular administration [115–117].

While being paramount for efficient TA delivery and expression, headgroup modifications can lead to mRNA instability [21].

##### Fusogenic properties

It is widely accepted that adopting a cone shape upon protonation in endosomal compartments facilitates endosomal escape [118]. Also, the incorporation of double bonds in the alkyl chain, increasing the degree of unsaturation, replacing alkene groups with ester bonds, adjusting alkyl chain length to the optimum, and using rigid linkers can contribute to the cone shape [115, 116, 118–121].

##### Metabolic behavior

For a patient to enjoy an effective therapeutic response from LNP-delivered mRNA vaccines, several doses of administration are required. One cannot achieve this amount of vaccine delivery if the vaccine induces considerable toxicity. A persistent theme in LNP vaccine development is the incorporation of biodegradable constituents into the LNPs to reduce off-target systemic toxicity [122]. Ester bonds in the alkyl chain are specifically of interest, which can be hydrolyzed easily by numerous intra- and extracellular lipases and esterases. The resulting by-products can be readily excreted or go through further metabolism [122]. One major hurdle associated with biodegradable materials like ester bonds, is that these compounds are quite vulnerable to the enzymes in the circulation after administration which can tamper with efficiency [116]. Other investigations are directed toward the modification of helper lipids, ionizable lipid ratio, and PEG molecular weight to further improve clinical responses [123, 124].

#### Polymer-based mRNA delivery systems

Polymer-based delivery systems tend to be less preferable compared to LNPs because of their lower purity, lower clearance rate, and higher toxicity [125, 126]. Polyethylenimine (PEI) is a type of cationic polymer commonly used as an mRNA delivery vehicle despite its low clearance rate, high toxicity, and low biodegradability. A PEI formulation has successfully induced immunity against the Influenza virus in mice by delivering a hemagglutinin-encoding SAM [29]. To overcome the limitations arising from toxicity, low molecular weight PEI with fatty chains has been developed to deliver both siRNA and antigen-encoding mRNA [127, 128].

Also, polysaccharides have been used rather extensively to deliver mRNA molecules in vivo and in vitro. Chitosan, a commonly used polysaccharide excipient, was used for the delivery of hemagglutinin-encoding SAM, and antigen expression in DCs was observed [129].

Polyamidoamines (PAMAM) have also been used for mRNA delivery. Khan et al. demonstrated the efficiency of fatty-chain modified PMAMs in the delivery of both siRNAs for lung cancer and antigen-encoding SAM for protection against Ebola, H1N1 influenza, and Toxoplasma gondii [130].

In order to increase the clearance rate and decrease charge-induced toxicity, biodegradable polymers such as poly (β-amino esters) (PBAEs), amino polyesters (APEs), and poly (CBA-co-4-amino-1-butanol) (pABOL) have been developed which are further discussed in [30, 131–134].

Charge-altering releasable transporters (CARTs) are another group of polymers that provide an innovative approach to mRNA delivery, addressing both problems of mRNA release and tolerability issues. Besides providing a mechanism for the release of mRNA molecules, transitioning from ester to amide groups makes CARTs acquire a more neutral charge, precluding toxicity issues [135–137]. These vaccines are usually administered intradermally. See Table 1.

#### Peptide-based mRNA delivery

Cationic peptides, especially protamine were used in many early studies for mRNA delivery. Protamines are cationic arginine-rich proteins that condense mRNA cargo as they protect it from external degrading factors. They can also act as adjuvants through interaction with TLR7/8 [138]. The main downside of this platform of vaccination is the tight junction between mRNA molecules and cationic peptides. To overcome this issue, RNActive^®^ has been developed by CureVac that combines naked antigen-encoding mRNA and protamine-mRNA complexes. This approach exploits the adjuvant effect of the protamine-mRNA complex and the antigen expression of the naked mRNA [21]. These vaccines are usually administered intradermally. See Table 1.

In a phase I clinical trial, a protamine-complexed RNActive vaccine encoding six TAs (NY-ESO-1, MAGE-C1, MAGE-C2, 5T4, surviving, and MUC1) was administered intradermally into patients with NSCLC combined with durvalumab (anti-PD1), with or without tremelimumab (anti-CTLA4). Although this trial has been completed, to our knowledge, no results have been published yet [139].

Cationic cell-penetrating peptides (CPPs) trigger micropinocytosis through the clustering of the negatively charged glycosaminoglycans on the cell surface [125]. A fusion peptide of truncated protamine and a short CPP called Xentry was used to deliver cystic fibrosis transmembrane regulator (CFTR) mRNA into human epithelial cells in vitro [140]. Investigating these CPPs to deliver TAs merits attention since they could enhance the resulting anti-tumor immune response.

### Viral-based mRNA cancer vaccination

Viral particles can be used in many ways to assist us in treating cancer. For example, some viral particles have tropism for malignant cells which are called oncolytic viruses. Some of these viruses include measles virus [141, 142], herpes simplex virus [143], and vesicular stomatitis virus [144]. Apart from their direct cytopathic effect on tumor cells, by promoting TA release, oncolytic viruses can enhance the immune-mediated clearance of tumor cells [145]. In this section, viral particles as vectors for mRNA vaccines will be discussed. To do this the antigen of interest is encoded in an mRNA molecule and internalized into a virus as a transgene. To ensure biosafety, virtually every potentially pathogenic gene is omitted from the viral particles [145]. Several viruses including adeno-associated viruses [146], flaviviruses [147], picornaviruses [148], lentiviruses [149], and alphaviruses [150] have been successfully used as mRNA delivery vehicles but not for cancer vaccination. Investigation into their application as mRNA delivery vehicles for cancer vaccines might broaden the scope of our understanding of the anti-tumor immunity and TME. All classes of mRNA molecules including nrRNA, SAM, and taRNA can be delivered into host cells using viral particles. Virus-loaded mRNA vaccines are usually administered intramuscularly. See Table 1.

Compared to other immunotherapies, recombinant viral vaccines are manufactured and administered more easily [145]. Each virus has its advantages and disadvantages with regard to its application as a vector for cancer vaccination. For instance, when used as a vector for vaccination, the Vaccina virus offers several advantages including easy manipulation in laboratory settings, natural immunogenicity which boosts antigen presentation to APCs, and strong elicited cellular and humoral immune responses [145]. Adenoviruses have low pathogenicity, high packaging capacity, tropism for different cell types, and no risk of insertional mutagenesis. Adeno-associated viruses (AAVs) have a good safety profile and broad tropism for different cells. They also confer a long-term gene expression which can reduce the number of required vaccine doses. Their main downside is their limited packaging size. Lentiviruses have the major risk of insertional mutagenesis which can lead to developing different cancers and genetic disorders. To overcome this issue, integrase-deficient lentiviruses have been developed which can mitigate these risks [151].

These vectors are cost-efficient and ‘off the shelf’ which makes them ideal for multi-center clinical trials [145]. These viral-based vaccines can be administered through a variety of routes, making them applicable for unconventional routes including intratumoral administration [145, 152]. All classes of mRNA molecules including nrRNA, SAM, and taRNA can be delivered into host cells using viral particles. Most viral vectors are strongly immunogenic attributed to the pro-inflammatory environment induced by the viral proteins, which can augment the resulting T-cell-mediated immune response. More importantly, many viruses have been shown to infect APCs with specific tropism for DCs which facilitate the process of immunogenicity against cancer antigens [153–158].

There are some reported difficulties regarding viral-based vaccines including integration into the host genome in DNA viruses, infectious risk, cytotoxicity, and immunogenicity of viral particles which can complicate multiple dosing [159]. Some approaches have been developed which could be used to overcome these issues. To address the problem of anti-viral neutralizing antibodies, prime-boost vaccination strategies have been developed in which, usually, the prime vaccination is done using a viral-vector vaccine while boost shots use other types of viral vectors or other delivery vehicles including but not limited to lipid-based vaccine, protein/peptide-based vaccines, and DC-based vaccines [145, 160–163]. Other approaches including ex vivo viral infection of APCs and inserting immunostimulant-encoding genes such as GM-CSF in the viral vector have also been investigated [145].

## Combination strategies

Even after the induction of an anti-tumor immune response, there are several boundaries that limit the efficiency of cancer vaccines such as the desmoplastic reaction seen in some solid tumors like pancreatic cancer [17]. As pointed out earlier, the main obstacle on the way of mRNA cancer vaccines to develop an effective anti-tumor response in the immunosuppressive nature of most of the tumors. Immunosuppression is a key regulatory component of the immune system as it harnesses it. This immunosuppression is crucial for self-tolerance and wound healing. Malignant cells can exploit this regulatory system to effectively paralyze the immune cells within their microenvironment to avoid detection and destruction. They do so by secreting immune-modulatory cytokines and recruiting inhibitory cells such as regulatory T-cells and tumor-associated macrophages (TAMs) to pathologically exaggerate the regulatory mechanisms. Numerous strategies have emerged to augment the immune response, some of which have been combined with mRNA vaccines to enhance their performance. For instance, PD-L1 and CTLA-4 are excessively expressed in some tumors to inhibit T-cells. Monoclonal antibodies against these proteins can prevent this inhibition and improve clinical responses [164, 165]. It is important to understand that these immune check blockers (ICBs) help the activated T-cells to exert their effect on tumor cells but tumor cells can create a TME so hostile that even antigen presentation to T-cells is disrupted. In other words, T-cells are not even activated to be potentiated by ICBs. Through the presence of aberrant APCs in the TME, the antigen presentation to T-cells is hindered. Even worse, some of these APCs promote tumor angiogenesis, metastasis formation, and immune suppression through the production of VEGF and TGF-β [166]. Targeting markers on the surface of these defective APCs such as colony-stimulating factor 1 (CSF1) can impair tumor growth [167, 168]. Targeting other immune-inhibitory cells in the TME such as myeloid-derived suppressor cells (MDSCs) and cancer-associated fibroblasts (CAFs) can grant similar results [169].

As described earlier, there is much controversy regarding the impact of type I IFN signaling on mRNA vaccine efficacy. Some studies indicate that increased innate immune response driven by mRNA and delivery vehicle, does not augment the immune response while other studies counterargue by demonstrating enhanced immune responses via combination with adjuvants such as TLR agonists and cytokines [117, 170–172]. RNActive is an example, which has been incorporated into numerous mRNA cancer vaccine trials. Enhanced anti-tumor immunity was achieved by combining naked unmodified mRNA with mRNA-protamine which acts as an adjuvant by stimulating TLR7/8 [173]. Stimulators of interferon genes (STING) agonists have also been used complementary to mRNA vaccines with encouraging results as reported by Miao and coworkers [119, 174].

Co-stimulatory ligands and receptors have especially been applied in mRNA-transfected DCs. Co-transfection with these ligands and receptors including but not limited to OX40L (OX40 ligand), ICOSL (Inducible T-cell costimulator ligand), CD70, caTLR4, GITRL (Glucocorticoid-induced TNF-related ligand), and 4-1BBL (4-1BB ligand) has shown varying degrees of success.

Rocapuldencel-T is a vaccine platform employing mature MoDCs co-loaded with both amplified tumor mRNA and CD40L-encoding mRNA [84, 175]. In a two-armed ADAPT phase III clinical trial, Rocapuldencel-T efficiency was evaluated in subjects with metastatic renal-cell carcinoma (mRCC). One arm was treated with Rocapuldencel-T plus standard of care treatment, while the second arm was treated with SoC alone. The trial’s primary objective was to evaluate and compare overall OS in the two arms in a median follow-up of 29 months. The vaccine failed to improve OS and PFS but it was determined that higher DC-produced IL-12 levels and higher numbers of regulatory T-cells prior to treatment were potential survival predictors in patients receiving DC-based immunotherapy [175].

TriMix is another mRNA platform that encodes three co-stimulatory molecules, CD40L, CD70, and constitutively active TLR4 (caTLR4). It has induced superior T-cell priming compared to transfection with other co-stimulatory and cytokine molecules [176]. In a single-arm study, TA-specific T-cell responses were evaluated in stage III or IV metastatic melanoma patients treated with Trimix-transfected T-cells and ipilimumab. Twelve out of fifteen patients showed meaningful T-cell responses in the enzyme-linked immunosorbent assay (ELISA) after in vitro T-cell stimulation with significantly broader and stronger immune responses in patients with partial or complete clinical responses [177].

Cytokines can also be incorporated into mRNA and co-transfected into tumor-antigen-presenting DCs to enhance DC maturation and T-cell priming. Cytokines such as GM-CSF, IL-12, and IL-15 are of most interest in this field [178–182]. The resulting activated DCs have enhanced ex vivo migratory capacity and the primed T-cells are more cytotoxic [178, 179]. Transfection of GM-CSF-encoding mRNA into TA-loaded DCs significantly increased the cytotoxicity of bulk splenocytes in CT26 tumor-bearing mice [178].

As will be discussed in the intratumoral injection section, co-stimulatory molecules can be injected solely into the tumors to modify the immunosuppressive microenvironment of the tumor to produce an effective anti-tumor immune response.

Although the results show tremendous advances compared to non-combined treatments, these combination strategies don’t seem to radically increase the effectiveness of mRNA cancer vaccines. One reason might be the absence of individualized tumor marker detection prior to combination therapy. By evaluating the patients and identifying tumor markers, one can specifically target them and potentially enhance the clinical outcomes. Also, further research is required to elucidate the physiologic effect of signaling molecules in the TME.

## Routes of administration

The vaccination route can remarkably impact the resulting immune response, making it a worthwhile target for modification [183]. The administration route is primarily chosen based on the targeted organ and the utilized delivery vehicle. For example, intranasal mRNA vaccine delivery can be used to treat lung cancer [184]. Also, for instance, LNP-loaded vaccines are usually administered intravenously, and naked mRNA vaccines are administered intranodally. See Table 1. The administration routes can be classified into conventional routes including intradermal (ID), intramuscular (IM), and subcutaneous (SC), and unconventional routes including intranodal, intratumoral, and intravenous (IV) routes.

### Intravenous

Systemic administration of naked mRNA is not a common theme in cancer vaccinology because of the high risk of systemic toxicity, the subjection of injected molecules to ubiquitous RNases, and conjugation with serum proteins resulting in a diminished internalization rate. Because of these limitations, systemically administered mRNA vaccines should be contained within a vehicle to be protected from degradation [185–188].

An important factor regarding systemic administration is that the optimization of the electrical charge of the synthetic vehicle is a crucial determinant of the vaccine’s efficacy. Positively charged cationic LNPs tend to aggregate in the liver which is not ideal for DC activation [189]. Using mRNA-lipoplexes, it has been elucidated that the lipid/mRNA ratio reflecting the net charge, plays an important role in the bio-distribution of the antigen-encoding mRNA as the negatively charged particles, predominantly target DCs in secondary lymphoid tissues [190]. These negatively charged particles induced an effective immune response in mouse models and entered clinical trials concerning advanced melanoma and triple-negative breast cancer.

### Intradermal and subcutaneous

Skin is an immunocompetent organ containing several kinds of APCs, making it an ideal site for vaccination. An early seminal study indicated that the intradermal injection of whole tumor mRNA can lead to diminished tumor growth in a fibrosarcoma mouse model [90]. The intradermal injection has been the principal route of the RNActive vaccine platform administration. In humans with castration-resistant prostate cancer, an RNActive vaccine encoding multiple prostate cancer-associated antigens elicited antigen-specific T-cell responses in the majority of the recipients [191]. For enhanced internalization of mRNA molecules, micro-projectile devices including Gene-gun and permeabilizing strategies including electroporation have been used [76, 192, 193].

In one clinical study, a personalized mRNA vaccine in combination with sintilimab (an anti-PD1 monoclonal antibody) is being evaluated in patients with hepatocellular carcinoma in an adjuvant setting following tumor resection. The vaccine encodes tumor neo-antigens and it is administered subcutaneously. To our knowledge, no results of the trial have been posted yet. (NCT05761717)

### Intramuscular

Among conventional options, intramuscular (IM) administration is more common owing to the flexibility of dosing volume, the ease of administration, feasibility, and less systemic and local administration-related side effects that are more associated with intradermal and subcutaneous injection [194]. Contrary to intravenous administration, intramuscular and intradermal administration is associated with a longer duration of antigen expression. This prolonged antigen exposure to the immune system can result in higher antibody titers [195]. One minor disadvantage of the intramuscular route is the relative immunologic poverty of muscle tissue. Skeletal muscles do not harbor many tissue-resident immune cells [196, 197]. So, initially, the bulk of antigen expression is carried out by myocytes which are not professional APCs. Eventually, however, the local inflammation of the muscle tissue causes recruitment of immune cells such as APCs which will further enhance antigen presentation. Incorporating elements such as adjuvants into the mRNA molecule which will further enhance the pro-inflammatory environment of the injection site will further improve the immune response [197].

In a phase I clinical study, an intramuscularly-administered personalized mRNA vaccine encoding tumor neo-antigens is being investigated in combination with pembrolizumab in patients with advanced solid tumors. The primary objective is to determine the safety and tolerability of the vaccine while the secondary objective is to evaluate CD4 + and CD8 + immune responses following vaccination. To our knowledge, no results of the trial have been posted yet. (NCT05916248)

### Intranodal administration

Intranodal administration is an unusual yet efficient way of vaccine delivery. Direct injection of naked mRNA into secondary lymphoid tissues, offers the advantage of targeting APCs in the site of T-cell activation and obviating the necessity of APC migration. Co-administration with FLT3L or TriMix can augment the resulting immune response [98–100]. Intranodal injection of naked mRNA encoding for the E7 protein of HPV-16 combined with TriMix protected a mouse model against an E7-expressing tumor [92]. The success of preclinical trials applying intranodal injection led to the initiation of clinical trials mostly concerning advanced melanoma [198] and hepatocellular carcinoma (HCC) [199].

In a clinical trial using the intranodal injection of DCs electroporated with mRNA encoding for tyrosinase and glycoprotein-100 (gp100), together with TriMix in patients with metastatic melanoma, limited clinical responses were observed [200].

Several studies have been conducted to determine the degree of immunogenicity of each of the administration routes. IM and SC are the two major administration routes for mRNA vaccine delivery because of their relative ease of administration, low systemic toxicity, and less invasive nature [21]. As shown by Ols et al., who investigated the impact of vaccination routes (mainly IM and SC) on antigen trafficking and immune response in Rhesus Macaques using fluorescently labeled HIV-1 envelope glycoprotein trimers displayed on liposomes, both SC and IM administration routes resulted in antigen uptake, APC migration and a nearly equal magnitude and quality of antigen-specific immune response [201]. Pardi et al. demonstrated that IM and ID administration result in a more prolonged antigen expression with higher half-life rates, while SC and IM administration result in a higher antigen expression magnitude [189]. A hot topic in mRNA vaccinology is the relative chronology of IFN signaling and TCR activation. It has been demonstrated that the closer IFN signaling and TCR activation are, the more potent the immune response is. This optimal time interval is best acquired in systemic administration through the IV route, precluding the detrimental effects of mRNA’s innate immunogenicity on the CTL-mediated immune response [190, 202].

In an ongoing phase I clinical trial, mRNA-2752, an LNP-based mRNA vaccine encoding OX40L, IL-23, and IL-36γ was administered intratumorally with or without intravenous ICB (Durvalumab or Tremelimumab). Interestingly, no TAA or TSA is encoded in this vaccine as it relies on the immune-modulatory effects of the encoded proteins. IL-23 and IL-36γ act synergistically to transform the immunosuppressive TME into a pro-inflammatory one by recruiting immune cells such as γδT-cells, natural killer (NK) cells, and APCs and revivify the pre-existing immune cells. OX40L can facilitate the expansion of CD4 + and CD8 + cells upon encounter with the TAs in the TME. Intratumoral administration optimally evokes the desired local effect of the vaccine. The primary objective of this trial is to evaluate the safety and tolerability of this vaccine and to define the maximum tolerated dose. To our knowledge, no results have been posted yet [203].

## Neoantigen encoding mRNA cancer vaccines

Neoantigens or tumor-specific antigens (TSA) are products of non-synonymous somatic mutations that could be targeted for personalized therapy. Apart from genomic mutations, neoantigens can derive from dysregulated RNA splicing, aberrant post-translational protein processing, and viral-encoded genes (as seen in nasopharyngeal cancer caused by EBV and cervical cancer by HPV) [204–207]. There are some limitations associated with the application of TAAs including: (A) the Limited number of TAAs for some solid tumors, (B) the Presence of TAAs in normal tissues (TAAs are not tumor-specific), and (C) Since TAAs are self-antigens, they are subject to central and peripheral tolerance. Fortunately, TSAs are not bound by these limitations and can be used to enhance enduring anti-tumor responses that can prevent disease recurrence [208].

In order to design these vaccines, firstly, tumor mutations should be detected. After tumor biopsy and whole-exome sequencing, non-synonymous mutations are detected through comparison with whole-exome sequencing of non-cancerous cells of the affected individual. Then TSAs are ranked based on their immunogenicity using in silico prediction algorithms and in vivo binding assays [202].

However, despite the advantages we mentioned, the process of identifying, selecting, and producing neoantigens is time-consuming, tiresome, and expensive. Only around 10% of non-synonymous mutations produce neoantigens with high affinity for MHC and about 1% of these are recognized by T-cells [209]Hopefully, with the current cross-integration between different branches of science such as immunology, biochemistry, computer science, and artificial intelligence, one should be optimistic about the future of neoantigen-based treatments [210, 211]. In a single-arm phase I clinical trial, IVAC-MUTANOME, a poly-neo-epitope-encoding naked mRNA targeting the unique mutation signature of individual patients was assessed for safety and tolerability upon repeated intranodal dosing and vaccine-induced cellular immune response in patients with stage III or IV melanoma. The vaccine was well-tolerated and neoantigen-specific CD4 + and CD8 + T-cell responses were detected in patients against multiple TSAs with considerable progression-free survival [201]. Other neoantigen-related trials have been discussed in the clinical applications section.

Tumors have developed several mechanisms to evade the immune response directed against neoantigens. The effect of the immune-suppressive TME was previously discussed. Loss of neoantigen can be achieved through several ways including copy number loss, repression of transcription, epigenetic modifications, and post-translational mechanisms. All of these routes, deprive the immune system of a valuable resource that could otherwise be used to contain tumor growth. These mechanisms have compelled researchers to incorporate several neoantigens in a vaccine to minimize the effects of antigen loss [209]. In a study by Rosenthal and colleagues, 43 out of 88 patients with NSCLC had evidence of neoantigen loss [212]. Another approach is the disruption of HLA heterozygosity and MHC downregulation to prevent antigen presentation. In a patient with colorectal cancer, who had seven lung metastases, all of the pulmonary lesions regressed shortly after the infusion of neoantigen-specific tumor-infiltrating lymphocytes (TILs); however, nine months later one of the lesions was found to have progressed. Upon further analysis of the excised lesion, it was determined that the haplotype on chromosome 6 which encodes the HLA-C*08:02 MHC-I molecule had been deleted. This meant that the neoantigen was no longer presented on tumor cells to be targeted by TILs [213].

## Clinical applications

Here we will discuss some of the most notable clinical and preclinical studies carried out regarding mRNA cancer vaccination in Table 1 and some studies have been discussed in more detail.

### DC-based mRNA vaccines

A phase I/II clinical trial was designed and carried out to evaluate the safety and immunogenicity of a whole-tumor mRNA-transfected DC vaccine in 22 patients with stage 3 or 4 melanoma. Autologous monocyte-derived DCs were developed using maturation and activation signaling molecules including GM-CSF, IL-4, IL-1β, IL-6, TNF-α, and prostaglandin-E2 (PGE2). Out of the 22 subjects, 12 received intranodal injections into the inguinal lymph nodes with ultrasound guidance while the other 10 patients received intradermal injections 10 centimeters below the inguinal ligament where the lymphatic drainage terminated in the same inguinal lymph nodes. Whole-tumor mRNA was obtained using tumor biopsy. Based on previous studies, it was decided to use all the mRNA samples available for each patient. mRNA concentration during the electroporation process had considerable variation among different patients but no significant difference in the resulting immune response was observed between patients on this ground. The vaccine was administered weekly in 4 doses.

The vaccine was generally well-tolerated with no treatment-related severe side effects. Limited side effects including local injection-site inflammatory reactions and flu-like symptoms were observed. One patient experienced tumor pain and one developed vitiligo. In patients receiving intranodal injections, an increase in the cortical/paracortical region width was observed, indicating immune-cell proliferation in these areas. The T-cell immune response was evaluated by T-cell proliferation assays, delayed-type hypersensitivity skin (DTH) tests, ELISpot assays, and Bioplex serum cytokine assays.

For the T-cell proliferation assay, PBMCs were harvested from the subjects before the vaccination, 3 weeks, and 10 weeks after the last vaccination. These cells were cultured and frozen. Also, T-cells were extracted from each patient before and after vaccination. Freshly thawed PBMCs were mixed with T-cells from before and after vaccination separately to compare the resulting T-cell counts between the two preparations. If the difference in T-cell counts of the two preparations was not indicative of antigen recognition by T-cells, patient-specific tumor-mRNA transfected DCs (tDCs) were added to the preparation once or twice to stimulate T-cell proliferation. This proliferation assay was performed on 19 patients, with 10 patients demonstrating positive results of which 3 were on freshly thawed PBMCs, 4 upon the first stimulation with tDCs, and 3 upon the second stimulation. Among these patients, 3 had positive pre-vaccination proliferation assays but after vaccination, the T-cell proliferation assay signified a boosting effect on the pre-existing anti-tumor immune response. The ELISpot assay roughly showed the same results. The T-cell proliferation assay on PBMCs collected 10 weeks after the vaccination, showed that a significant tDC-specific response can be detected in 4 out of the 6 evaluable patients indicating a considerable endurance of the tumor-specific immune response. Serum cytokine analysis revealed the production of IL-2, IFN-γ, TNF-α, IL-5, and MIP-1 among other cytokines.

In the DTH skin test, tDCs, and mockDCs (DCs that are not electroporated with tumor-antigen encoding mRNA) were injected into separate sites after the completion of vaccination. DTH results were measured as the diameter of the injection site erythema 48 h after the test injection. The test results were interpreted as the difference between the diameter of erythema between the tDC and mockDC injection sites. Five patients showed moderately/strongly positive skin tests while 3 patients exhibited a weakly positive result.

Overall it could be interpreted that 10/22 subjects developed immunological activity against TAs, 10 were non-responders and the other 2 were inconclusive. Also, the results did not point to a meaningful immunological difference between intradermal and intranodal routes of administration. From the time of vaccination, the mean survival of the subjects was 12.3 months. Patients, who survived for more than 20 months after vaccination, all belonged to the group of immunological responders. Because of the absence of any control group in this non-randomized trial, the results were compared to the age-matched advanced stage 4 melanoma patients in the same hospital who did not receive the vaccine immunotherapy. The patients in the trial showed more survival time, both from the time of vaccination and from the time of disease progression. Ten weeks after the vaccination, the majority of trial subjects (18/22) showed progressive disease, 2 showed stable disease and 2 were not evaluable [214]. This shows that either the stimulated immune response was short-lived or the tumor has developed resistance. Further investigation into the antigens involved in the anti-tumor response might be helpful for future target selection.

In another phase IB clinical study, autologous MoDCs cultured in GM-CSF, IL-4, and autologous plasma and co-electroporated with mRNA molecules encoding melanoma-associated antigens (MAAs) including MAGE-A3, MAGE-C2, tyrosinase and gp100, linked to an HLA class II targeting signal were injected intradermally and intravenously into pretreated patients with unresectable, histologically confirmed stage 3 or 4 melanoma, who did not receive any other cancer-related treatment at the time of the trial. The subjects were allocated into 4 cohorts based on vaccine dosage and received five doses of the cancer vaccine with the first four received biweekly and the last one received ten weeks after the fourth dose.

Limited treatment-related side effects including chills, skin irritation, erythema, edema, fever, and flu-like symptoms were noted, probably due to the cytokine release phenomenon that can be an indicator of efficient immune system activation. Tumor response was evaluated by RECIST (Response evaluation criteria in solid tumors). Two complete responses and two partial responses were observed among the initial 15 subjects. Additionally, 4 patients experienced stable disease following the vaccine therapy. The median PFS and OS were 5 and 14 months respectively.

For T-cell response evaluation, a skin biopsy acquired sufficient skin-infiltrating T-cells (SKILs) in 10 out of 13 tested patients, one week after the fourth dose of the vaccine. Among these patients, treatment-specific CD8 + T-cells were observed in 4 individuals. Among patients without sufficient extracted SKILs, 2 patients revealed treatment-specific CTLs after in vitro re-stimulation. In 5 out of 12 patients, CD4 + T-cells were observed with specificity for treatment-related TAs. In serum cytokine analysis, a significantly higher concentration of IL-1β and IL-6 were observed compared to pre-administration concentrations. A moderate increase in pro-inflammatory cytokines and chemokines such as IFN-γ, TNF-α, IL-8, and macrophage inflammatory protein (MIP-1) was also noted.

Patients in all dose cohorts experienced anti-melanoma response but a definite conclusion regarding the optimal dosage remains elusive due to the inadequate sample size. The vaccination proved to be safe, feasible, and immunogenic, but further modifications are warranted to reach the efficiency required for routine clinical application [82].

An open-label, single-arm, phase II clinical trial evaluated the impact of autologous MoDCs co-electroporated with mRNA molecules coding for four MAAs (MAGE-A3, MAGE-C2, tyrosinase, and gp100) and TriMix (CD40L, CD70, and caTLR4) combined with ipilimumab on 39 pretreated patients with unresectable histologically confirmed stage 3 or 4 melanoma. The patients were followed for a median of 5 years. An equal ratio of DCs expressing each MAA was used in the vaccine development. The vaccine was administered four times intravenously and intradermally along with ipilimumab every 3 weeks. Since ipilimumab was the only FDA-approved ICB drug for patients with advanced melanoma at the initiation of the trial, PD-1 blockers were further evaluated in the following trials for combination therapy in melanoma patients as they have shown more safety and efficacy. Grade 3 or 4 immune-related adverse events were reported in 36% of the patients but grade 5 adverse events were not reported.

Immune monitoring was carried out in 15 subjects with pre-vaccination (prevac) and post-vaccination (postvac) PBMC samples, using intracellular cytokine staining (ICS), ELISpot, ELISpot after in vitro T-cell stimulation (IVS-ELISpot), ICS after in vitro T-cell stimulation (IVS-ICS) and TCR repertoire sequencing after in vitro T-cell stimulation (IVS-TCR_seq_). Twelve out of fifteen subjects were immune responders according to the IVS-ELISpot assay, among which 10 subjects responded to more than one MAA and 3 responded to every single MAA in the vaccine composition. ICS revealed IFN-γ, TNF-α, and IL-2 production. IVS-ICS indicated predominant CD8 + activation. IVS-TCR_seq_ introduced tyrosinase as the most immunogenic MAA. A weak CD4 + response was detected in only 2 patients, although it should be kept in mind that MAAs were linked to lysosomal-associated membrane protein (LAMP), an HLA-class II targeting signal (DC-LAMP). In the postvac samples, a significantly higher number of T-regs were detected with a CD3+, CD4+, CD127_low_, CD25_high,_ and Foxp3_high_ expression profile. Also, significantly higher CD62L expression was detected in postvac T-regs compared to prevac T-regs. This abundance of T-regs in postvac samples can be explained by the IL-2 production by the activated CD8 + lymphocytes. Since postvac samples unveiled major immune activation, the T-reg response must have been not so effective in immune suppression, although it could have had a dampening effect on the resulting tumor-specific T-cell immune response.

After more than five years of follow-up, the overall survival of the participants was around 28% and progression-free survival was seen in 7 patients (roughly 18%) who were apparently cured. The OS is significantly higher compared to patients treated with ipilimumab alone. Furthermore, the clinical response seems to be tightly correlated with the immune response. Significantly higher numbers of cytokine-producing T-cells are observed in patients with complete response (CR) and partial response (PR) compared to patients with stable disease (SD) and progressive disease (PD). All patients with CR or PR were positive for at least two MAAs in the IVS-ELISpot assay. Also, T-cells in CR and PR patients had higher multi-functionality, as they mostly produced more than 2 kinds of cytokines in the immune-monitoring assays [177]. It might be useful for future trials to investigate the reasons for this heterogeneity in immunological and clinical responses between different patients. Also, a more personalized approach may be warranted as using the same treatment for each patient might not result in the best outcomes.

In a phase I clinical trial, 22 patients with stage IV metastatic melanoma with evidence of progressive disease and visceral involvement were treated with autologous MoDCs transfected with whole-tumor mRNA. Nine additional patients entered the trial as an expansion cohort, receiving the same treatment plus IL-2 injection. Each patient received 4 weekly doses of the vaccine either intradermally or intranodally into the inguinal region, followed by optional booster vaccination every month thereafter. IL-2 was injected intranodally 1–3 days after the fourth dose of the vaccine. Tumor mRNA was extracted from a metastatic lesion in every patient. Minor side effects including flu-like symptoms, tumor pain, and injection site inflammation were observed in most patients, but no severe systemic toxicity was observed. One patient developed vitiligo following vaccination.

Vaccine-induced T-cell responses were evaluated using T-cell proliferation assays and IFN-γ ELISpot. In the T-cell proliferation assay, T-cells from pre- and post-vaccination blood samples of the patients were incubated with either transfected DCs (tDCs) or non-transfected DCs (nDCs) and the results were compared between these two incubations to confirm a vaccine-specific immune response. A delayed-type hypersensitivity assay was also performed following the last dose of the vaccine.

According to T-cell assay results, 16 patients responded to the vaccine, 12 were non-responders and 3 had inconclusive results. Post-vaccination T-cells extracted from the majority of patients also indicated a proliferative response to nDCs, which can point to a possible epitope scattering following vaccine-mediated tumor lysis. T-cells were poly-functional secreting a vast spectrum of cytokines including Th1-associated cytokines (IFN-γ, TNF-α), Th2-associated cytokines (IL-5, 1 L-13), and even Treg-associated cytokines (IL-10). IFN-γ/IL10 ratio was significantly variable among different patients, probably affecting the immune and clinical responses. Also, 6 patients had a moderately/strongly positive DTH result, all of whom had transfected-DC (tDC)-specific T-cell responses. Six additional patients had a weakly positive DTH response, 3 of which had a tDC-specific immune response in T-cell assays. These results suggest a correlation between the results of T-cell assays and the DTH assay.

The results denote a more potent immune response in patients undergone intradermal vaccine administration. Among those with intradermal dermal vaccination, 80% developed a vaccine-induced immune response compared to 38% in those with intranodal administration. Several hypotheses to elucidate the reason for this phenomenon were suggested, one of which states that the in vitro DC maturation may not result in functionally optimal cells, and the DCs injected i.d. may receive further maturation signals during migration. Finally, the successfully migrating DCs after i.d. vaccination will be a selected DC population, whereas the functionally immature reaching the lymph node after i.n. injection, may not optimally stimulate a vaccine response.

The overall survival of patients solely receiving the vaccine was 10 months compared to 13 months in those with additional IL-2 injections. Most of the patients retained the progressive disease and after ten years of follow-up, only 2 patients were alive. One of them developed complete remission following vaccination with recurrence three years later which responded well to ipilimumab (an anti-CTLA4 monoclonal antibody). The other patient had a partial response to vaccination but he developed a progressive disease after 5 years. He reached a stable disease after treatment with ipilimumab and pembrolizumab (an anti-PD1 monoclonal antibody). These results indicate that cancer vaccines and ICBs can have a synergistic effect.

In a meta-analysis, the patients in this trial had considerably longer overall survivals compared to melanoma patients receiving the conventional treatment adjusted for sex and metastatic category. OS was substantially longer in immune responders compared to non-responders, implying a correlation between the immune and clinical responses. Interestingly, all patients with an OS of higher than 20 months were immune responders. The vaccine also proved to be more efficacious in patients with a lower tumor burden [215].

Immune responses were evaluated using ELISA for the detection of immunizing antibodies against PA2024 and T-cell proliferation assays, both of which pointed to a significantly stronger anti-tumor immune response in the vaccine group. After a median follow-up period of 34 months, the survival rate was 38.4% in the Sipuleucel-T subgroup compared to 30% in the placebo subgroup. Median survival was 4.1 months longer in the Sipuleucel-T group compared to the placebo group. Time to objective disease progression was nearly the same in both arms, probably due to the delayed onset of anti-tumor responses after active immunotherapy. In placebo patients receiving the vaccine immunotherapy after the trial, the median survival was nearly 12 months longer compared to those who did not. The vaccination did not preclude any future treatments including chemotherapy [216]. As mentioned, the vaccine improved the survival rate, but not significantly. To overcome this issue, the inclusion of some TSAs in the vaccine might be beneficial, as the tumor might gain resistance to the treatment by loss of antigen.

In a phase I clinical trial, 11 patients with newly diagnosed stage IV glioblastoma received intradermal injections of pp65-LAMP mRNA-pulsed DC vaccine mixed with GM-CSF along with concomitant dose-intensified chemotherapeutic agent temozolomide (DI-TMZ). Human cytomegalovirus (CMV) proteins including pp65 are expressed in more than 90% of glioblastoma tumors while the surrounding brain tissue is usually negative for CMV-associated protein expression. Positive pp65 tumor staining was not included as an eligibility criterion for enrollment. Each patient received at least 3 doses of the vaccine. Reactive homeostatic proliferation following lymphopenia-inducing TMZ treatment can augment the vaccine-induced antigen-specific immune response. To get a better picture of this specific treatment’s efficacy, historical controls matched for other characteristics were utilized for comparison.

No adverse events were recorded except for a severe reaction to GM-CSF in one patient that ceased following the discontinuation of GM-CSF in future vaccines for this patient. IFN-γ ELISpot assays demonstrated an increase in response against pp65 following vaccination. Tetramer analysis of 6 patients indicated an increase in tumor-reactive T-cell population in all of them. Further analysis indicated an increase in the Treg population among CD4 + T-cells following vaccination. Despite this response, CD8 + T-cell responses also rose with an increased Treg/CTL ratio post-vaccination.

Significantly increased OS and PFS were observed compared to matched historical controls. A median survival gain of 30 months was achieved compared to controls. Four out of eleven patients remained progression-free for 59–64 months following vaccination, all of whom received at least four doses of the vaccine, signifying the dose dependency of the clinical response [217]. Overall, this vaccine proved to be safe and efficacious, and further investigations into its optimization are warranted.

### Naked mRNA vaccines

In a phase, I clinical study, intranodal administration of naked mRNA under ultrasonography guide was evaluated in patients with stage 3 or 4 melanoma with a history of recurrent disease or at a high risk of relapse. Neo-antigens were detected using comparative exome sequencing and the most immunogenic epitopes were chosen using antigen ranking based on the prediction of binding affinity to MHC I or II molecules and the expression rate of the mutation-encoding mRNA. Ten mutations were chosen for each patient and encoded into two mRNA pentatope RNAs. The median time from mutation selection to vaccine production was 103 days. Approaches to shorten this period of time might improve patient outcomes. The vaccine was well-tolerated without any significant vaccine-related side effects.

The immune response was evaluated mainly using IFN-γ ELISpot, performed on pre- and post-vaccination CD4 + and CD8 + T-cells obtained from blood samples with and without in vitro stimulation with RNA-transfected autologous PBMCs. Responses were detected against 60% of the predicted epitopes, two-thirds de-novo responses, and one-third augmented pre-existing responses. Each patient developed antigen-specific T-cell responses against at least three neo-epitopes. The majority of responses were solely CD4 + mediated while around a quarter of responses demonstrated concurrent CD4 + and CD8 + immune responses.

In a fraction of patients, MAA-encoding mRNAs were co-administered along with neo-epitopes. The immune response was far greater against neo-antigens probably owing to the lack of central immune tolerance. There was a significant reduction in recurrent metastatic events following vaccination. Eight patients had no radiologically detectable lesions at the start of vaccination. They remained recurrence-free through the entire follow-up period. Five patients had relapses shortly after inclusion and had progressing disease at the start of vaccination. Out of these patients, one had a complete response of tumor lesions unresponsive to local radiotherapy and CTLA-4 blockade. One had a complete response following the co-administration of PD-1 blockers. One had a partial response of abdominal lymph nodes, one had stable disease and one had mixed responses [93].

In another phase I/II trial, 30 patients with metastatic renal cell carcinoma (mRCC) received intradermal injections of unmodified in vitro transcribed mRNA encoding for six different TAAs (MUC1, CEA, Her2/neu, telomerase, survivin, MAGE-A1) along with adjuvant subcutaneous GM-CSF injections. All of the subjects had previously gone through nephrectomy. The patients were classified into two cohorts: A: 14 patients with intradermal vaccination on days 0, 14, 28, and 42, and B: 16 patients with an intensified protocol consisting of injections on days 0–3, 7–10, 28, and 42 with a higher dose compared to cohort A. All patients were vaccinated monthly thereafter until disease progression.

The vaccine proved to be safe with minor side effects, although one allergic reaction to GM-CSF was observed. Half the subjects developed stable disease for 3 months. The median survival was increased in the vaccination group compared to those receiving the SoC treatment. Also, median survival was significantly higher in immune responders compared to those without vaccine-induced immune response (89 months compared to 14 months). Patients in cohort B displayed a greater median survival compared to those in cohort A, signifying the enhanced efficacy of the intensified treatment. Five patients were still alive after a long-term follow-up of 9 years.

Immunological reactivity was studied in 20/30 subjects using IFN-γ ELISpot and chromium release assay with a 75% response rate among the evaluated subjects. Also, the immune response was correlated with a clinical benefit, as described earlier. It should be noted that a considerable fraction of the patients received tyrosine-kinase inhibitors, mTOR inhibitors, radiotherapy, or chemotherapy post-vaccination. All of these treatments exert an effect on patient survival in follow-ups, obscuring our view on the sole effect of treatment with the cancer vaccine [218]. It might be practical to evaluate the effect of naked mRNA vaccination without these confounding factors to be able to compare their efficiency with other platforms of mRNA cancer vaccines.

### Nanoparticle-based mRNA vaccines

In a phase II open-label clinical trial, an RNactive-based vaccine (BI1361849) was evaluated in 26 patients with stage 4 NSCLC with PR or SD after first-line chemotherapy or EGFR-TK (epidermal growth factor receptor- tyrosine kinase) blockage therapy. As previously discussed, RNA molecules can have adjuvant effects only when complexed with another molecule; but this inhibits translation and antigen expression. To overcome this challenge, protamine-complexed mRNA has been combined with naked mRNA to achieve both adjuvant effects and antigen expression. The subject should have had at least one tumor lesion eligible for local radiation and at least another measurable region for assessment of the radiotherapy effect to enter the trial. The mRNA molecule encoded six NSCLC-associated antigens including New York esophageal squamous cell carcinoma 1 (NY-ESO-1), MAGE-C1, MAGE-C2, survivin, 5T4, and mucin short variant S1 (MUC-1). The vaccine was injected intradermally. The patients were allocated to three arms based on the molecular and pathological characteristics of the tumor and the previous and current treatments. Each patient received a dose of the vaccine on days 1 and 8, along with radiation therapy on days 9 to 12 (4 × 5 Gy). After that, patients in arms 1 and 3 received a vaccine dose on days 15, 36, and 57, while patients in arm 2 received the same treatment on days 15, 29, 43, and 57. After that every subject received injections every 3 weeks for 6 months, and every 6 weeks thereafter until disease progression or toxicity.

Vaccine or radiation-related adverse events of grade 3 or higher were observed in 4/26 patients including fatigue, pyrexia, and dysphagia. Minor symptoms including injection-site reactions and flu-like symptoms were observed in a minute fraction of the subjects. The cellular immunity was evaluated by ICS and ELISpot assays and humoral immunity was evaluated by ELISA and serum profiling using an NSCLC-specific antigen array. Flow cytometry was used to separate antigen-specific T-cells from the mixture. Extracted PBMCs were treated with short peptides encoding each vaccine-targeted antigen. ICS was carried out on antigen-specific T-cells for IFN-γ, TNF-α, IL-2, and CD107a (LAMP-1) translocation in CD4 + and CD8 + T-cells which is a marker for T-cell degranulation and cytokine release. Of the 25 evaluable subjects, 21 demonstrated antigen-specific immune responses, of which 13 patients had immune responses against more than one vaccine antigen. Ten subjects had cell-based immunity according to ICS or ELISpot, and twenty showed anti-tumor IgM or IgG immunity. The number of antigen-specific T-cells increased over time with CD4 + T-cells responsible for most of this rise. The majority of T-cells were mono-functional according to ICS, meaning that they produced one major cytokine. Serum profiling was done using an NSCLC-specific antigen array which evaluated the humoral response against 32 known NSCLC-specific antigens to evaluate the effect of radiotherapy in inducing a cascade to broaden the anti-tumor immune response. In half of the subjects, tumor-specific antibodies against antigens not incorporated into the vaccine were detected, although some had these antibodies even before vaccination. Twelve out of 26 patients had stable disease and one showed PR, but this subject received concomitant pemetrexed treatment. Shrinkage of the non-irradiated lesion(s) was observed in 6 patients. The median PFS was 2.87 months and the median OS was 13.95 months [173]. As mentioned earlier, understanding the physiology of different responses between different patients might be crucial for selecting the right treatment for them. Also, using a personalized approach might be more efficient.

In an exploratory interim analysis of a phase I clinical trial, the immune and clinical responses to an intravenously administered nanoparticle liposome RNA vaccine or RNA-lipoplex vaccine (RNA-LPX) were evaluated. The RNA encodes four prevalent melanoma-associated antigens with high immunogenicity: NY-ESO-1, MAGE3, tyrosinase, and transmembrane phosphatase with tensin homology (TPTE). The drug was tested on patients with stage III or IV patients with resected or unresected melanoma expressing at least one of the TAAs mentioned above. Each patient received 8 doses of the vaccine every seven weeks with 3 + 3 dose escalation, followed by optional continued monthly vaccination. Three expansion cohorts were added receiving the same treatment with or without anti-PD1 antibodies. Mild to moderate transient flu-like symptoms were observed but no severe toxicity was reported. Exploiting the enhanced glucose uptake following TLR stimulation, a group of patients underwent PET/CT scans before and after vaccination, demonstrating increased uptake in lymphoid tissues, especially the spleen, shortly after vaccination.

The adjuvanticity of the vaccine was evaluated using serum cytokine analysis before and after vaccination, demonstrating increased IFN-α, IFN-γ, IFN-inducible protein (IP10), and IL12-P70 after vaccination. IFN-gamma ELISpot demonstrated a T-cell-specific immune response against at least one TAA in 75% of the patients without IVS. In 20 other patients, a 100% response was detected following IVS with autologous DCs loaded with TAAs. The responses were mostly CD4 + mediated and poly-epitopic. ICS indicated CD8 + cells were mostly PD1+/CCR7-/CD27+. T-cells increased in frequency over time in patients with monthly maintenance vaccination. T-cells were not affected by dose, presence of measurable disease at baseline, or combination with anti-PD1. Upon the transfection of T-cells with TCR clones obtained from antigen-specific T-cells, these cells were able to kill TAA-expressing melanoma cell lines.

42 patients were assessed for clinical responses, almost all of whom had previously undergone at least one ICB treatment. In patients who solely received the vaccine, 1 complete metabolic remission in PET/CT, 3 partial responses, and 7 stable diseases were observed. Out of the 17 patients who received concomitant vaccination and anti-PD1 therapy, 6 showed partial responses. Regression was mostly observed among the patients who received the highest dose of the vaccine along with anti-PD1 therapy. Tumor response was directly correlated with the tumor burden. Patients with the best clinical responses had a more robust and diversified T-cell response. The T-cell response was sustained in patients who received the vaccine for at least one year.

Interestingly two patients responded to the re-challenge with anti-PD1 therapy following vaccination. After anti-PD1 failure, these patients underwent vaccination, following which anti-PD1 was effective in causing tumor regression. CD8 + T-cells from these patients were mostly PD1 + which predicts a good augmentation effect following the administration of anti-PD1 therapy. Although ICBs are mostly effective in tumors with a high burden of mutation, this trial demonstrated that the co-administration of PD1 blockers can enhance the immune response in patients receiving TAA-loaded vaccines too [219].

## Conclusion

As the understanding of the biology of cancer grows, researchers have begun to appreciate the role of mRNA as a valuable asset in cancer immunotherapy. Although several obstacles lie in the way of their development, mRNA cancer vaccines are one of our most promising solutions for managing malignancies. Despite their current insufficiency to induce a clinically substantial immune response, these vaccines have shown their potential in numerous clinical trials and merit future investments. Several strategies are currently under investigation to fill in the gaps and form a more powerful and precise treatment. Optimizations regarding TA selection based on predicted immunogenicity, delivery vehicles, combination strategies, and routes of administration require further clinical research, and reviews like this can potentially broaden our view of the platform and provide a more comprehensive outlook on the opportunities of growth for mRNA cancer vaccines.

Future perspectives of mRNA cancer vaccines are most probably reliant on the progress of biomedical sciences. Focusing the attention towards neoantigen-based mRNA cancer vaccines is inevitable. There is simply too much inter-personal heterogeneity between different patients to consider a similar treatment for them as optimal. The emergence of next-generation sequencing and whole exome sequencing changed this narrative and made it possible to take individual markers into account before starting a treatment. This has already shown its supremacy over conventional treatments. To further build up this supremacy, finding more markers to evaluate each patient is necessary. For example, understanding more about the type of immunosuppressive cells in the TME, the surface markers of tumor cells and cancer-associated fibroblasts, and even serum tumor markers can help to specify our course of treatment to target the core of the problem. Each of these personal evaluations provides new drug targets to be used in combination with mRNA cancer vaccines which can have additive or even synergistic effects. Discovering the markers will not only determine the content of our treatment but also its dosing, the vector, and the route of administration. This kind of personal treatment will not only improve clinical outcomes, but will also alleviate the unpleasant side effects of the non-selective conventional treatments on the already in-pain cancer patient. Personalized therapy may seem to be too expensive and non-cost-efficient to be used for each cancer patient, but it seems to be the only option available to maximize the effectiveness of cancer treatments. Hopefully, in the near future, the widespread application of these mRNA cancer vaccines will be witnessed in clinical settings as a revolutionary treatment, saving millions of lives.

### Key points

- mRNA cancer vaccines are one of our most promising solutions for managing malignancies.
- The emergence of next-generation sequencing and whole exome sequencing changed this narrative and made it possible to take individual markers into account before starting a treatment, one instance of which being the neo-antigen-encoding mRNA vaccines.
- mRNA cancer vaccines are stealing the spotlight, especially after the huge success of mRNA-based COVID-19 vaccines.

## Data Availability

No datasets were generated or analysed during the current study.

## References

[1] Maruggi G, et al. mRNA as a transformative technology for Vaccine Development to Control Infectious diseases. Mol Ther. 2019;27(4):757–72. 30803823 10.1016/j.ymthe.2019.01.020 PMC6453507

[2] Li Y, et al. mRNA vaccine in cancer therapy: current advance and future outlook. Clin Transl Med. 2023;13(8):e1384. 37612832 10.1002/ctm2.1384 PMC10447885

[3] Sayour EJ, et al. Cancer mRNA vaccines: clinical advances and future opportunities. Nat Rev Clin Oncol. 2024;21(7):489–500. 38760500 10.1038/s41571-024-00902-1

[4] Riedel S. *Edward Jenner and the history of smallpox and vaccination*. in *Baylor University medical center proceedings*. 2005. Taylor & Francis. 10.1080/08998280.2005.11928028 PMC1200696 16200144

[5] Ni LJV. Adv mRNA-Based Cancer Vaccines. 2023;11(10):1599. 10.3390/vaccines11101599 PMC10611059 37897001

[6] He Q, et al. mRNA cancer vaccines: advances, trends and challenges. Acta Pharm Sin B. 2022;12(7):2969–89. 35345451 10.1016/j.apsb.2022.03.011 PMC8942458

[7] Tan T, et al. mRNA vaccine - a New Cancer Treatment Strategy. Curr Cancer Drug Targets. 2023;23(9):669–81. 36809966 10.2174/1568009623666230222124424

[8] Haq HN et al. *Pfizer-BioNTech (BNT162b2), Moderna (mRNA-1273) COVID-19 mRNA vaccines and hypersensitivity reactions.* 2022. 10.1016/j.jnma.2022.08.003 PMC9613973 36511275

[9] Kaznadzey A et al. BNT162b2, mRNA-1273, and Sputnik V vaccines induce comparable immune responses on a par with severe course of COVID-19. 2022. 13: p. 797918. 10.3389/fimmu.2022.797918 PMC9044856 35493476

[10] Chen J, Chen J, Xu Q. Current developments and challenges of mRNA vaccines. Annu Rev Biomed Eng. 2022;24:85–109. 35231177 10.1146/annurev-bioeng-110220-031722

[11] Fan C, et al. Cancer/testis antigens: from serology to mRNA cancer vaccine. Semin Cancer Biol. 2021;76:218–31. 33910064 10.1016/j.semcancer.2021.04.016

[12] Chehelgerdi M, Chehelgerdi MJMC. The use of RNA-based treatments in the field of cancer immunotherapy. 2023. 22(1): p. 106. 10.1186/s12943-023-01807-w PMC10401791 37420174

[13] Rock KL, Reits E, Neefjes J. Present yourself! By MHC class I and MHC class II molecules. Trends Immunol. 2016;37(11):724–37. 27614798 10.1016/j.it.2016.08.010 PMC5159193

[14] Wang B, et al. Recent advances in mRNA cancer vaccines: meeting challenges and embracing opportunities. Front Immunol. 2023;14:1246682. 37744371 10.3389/fimmu.2023.1246682 PMC10511650

[15] Jahanafrooz Z et al. *Comparison of DNA and mRNA vaccines against cancer.* 2020. 25(3): pp. 552–560. 10.1016/j.drudis.2019.12.003 PMC7080609 31843577

[16] Pandya A, et al. Future cancer Immunotherapy: DNA Vaccines Lead way. 2023;40(7):200. 10.1007/s12032-023-02060-3 PMC10251337 37294501

[17] Huang X, et al. Personalized pancreatic cancer therapy: from the perspective of mRNA vaccine. Mil Med Res. 2022;9(1):53. 36224645 10.1186/s40779-022-00416-w PMC9556149

[18] Liu C, et al. mRNA-based cancer therapeutics. Nat Rev Cancer. 2023;23(8):526–43. 37311817 10.1038/s41568-023-00586-2

[19] Rosa SS, et al. mRNA vaccines manufacturing: challenges and bottlenecks. Vaccine. 2021;39(16):2190–200. 33771389 10.1016/j.vaccine.2021.03.038 PMC7987532

[20] Perenkov AD et al. *In* Vitro transcribed RNA-Based platform vaccines: past, Present, and Future. Vaccines (Basel), 2023. 11(10). 10.3390/vaccines11101600 PMC10610676 37897003

[21] Miao L, Zhang Y, Huang L. mRNA vaccine for cancer immunotherapy. Mol Cancer. 2021;20(1):1–23. 33632261 10.1186/s12943-021-01335-5 PMC7905014

[22] Liu J et al. Cancer vaccines as promising immuno-therapeutics: platforms and current progress. 2022. 15(1): p. 28. 10.1186/s13045-022-01247-x PMC8931585 35303904

[23] Miao L, Zhang Y, Huang L. mRNA vaccine for cancer immunotherapy. Mol Cancer. 2021;20(1):41. 33632261 10.1186/s12943-021-01335-5 PMC7905014

[24] Flemming A. Self-amplifying RNA in lipid nanoparticles: a next-generation vaccine? Nat Rev Drug Discovery. 2012;11(10):749–749. 23023675 10.1038/nrd3854

[25] Rayner JO, Dryga SA, Kamrud KI. Alphavirus vectors and vaccination. Rev Med Virol. 2002;12(5):279–96. 12211042 10.1002/rmv.360

[26] Zimmer G. RNA replicons-a new approach for influenza virus immunoprophylaxis. Viruses. 2010;2(2):413–34. 21994644 10.3390/v2020413 PMC3185613

[27] Lundstrom K. Self-replicating RNA viruses for RNA therapeutics. Molecules. 2018;23(12):3310. 30551668 10.3390/molecules23123310 PMC6321401

[28] Dailey GP, Crosby EJ, Hartman ZC. Cancer vaccine strategies using self-replicating RNA viral platforms. Cancer Gene Ther, 2022: pp. 1–9. 10.1038/s41417-022-00499-6 PMC9275542 35821284

[29] Vogel AB, et al. Self-amplifying RNA vaccines give equivalent protection against influenza to mRNA vaccines but at much lower doses. Mol Ther. 2018;26(2):446–55. 29275847 10.1016/j.ymthe.2017.11.017 PMC5835025

[30] Blakney AK, et al. Big is beautiful: enhanced saRNA delivery and immunogenicity by a higher molecular weight, bioreducible, cationic polymer. ACS Nano. 2020;14(5):5711–27. 32267667 10.1021/acsnano.0c00326 PMC7304921

[31] Geall AJ, et al. Nonviral delivery of self-amplifying RNA vaccines. Proc Natl Acad Sci. 2012;109(36):14604–9. 22908294 10.1073/pnas.1209367109 PMC3437863

[32] Beissert T, et al. A trans-amplifying RNA vaccine strategy for induction of potent protective immunity. Mol Ther. 2020;28(1):119–28. 31624015 10.1016/j.ymthe.2019.09.009 PMC6953774

[33] Bahl K, et al. Preclinical and clinical demonstration of immunogenicity by mRNA vaccines against H10N8 and H7N9 influenza viruses. Mol Ther. 2017;25(6):1316–27. 28457665 10.1016/j.ymthe.2017.03.035 PMC5475249

[34] Richner JM, et al. Modified mRNA vaccines protect against Zika Virus infection. Cell. 2017;169(1):176. 28340344 10.1016/j.cell.2017.03.016

[35] Pardi N, et al. Zika virus protection by a single low-dose nucleoside-modified mRNA vaccination. Nature. 2017;543(7644):248–51. 28151488 10.1038/nature21428 PMC5344708

[36] Hu C, et al. Amplifying mRNA vaccines: potential versatile magicians for oncotherapy. Front Immunol. 2023;14:1261243. 37936701 10.3389/fimmu.2023.1261243 PMC10626473

[37] Kenoosh HA, et al. Recent advances in mRNA-based vaccine for cancer therapy; bench to bedside. Cell Biochem Funct. 2024;42(2):e3954. 38403905 10.1002/cbf.3954

[38] Nagorsen D, Thiel E. HLA typing demands for peptide-based anti-cancer vaccine. Cancer Immunol Immunother. 2008;57:1903–10. 18317754 10.1007/s00262-008-0493-6 PMC11030559

[39] Linares-Fernández S, et al. Tailoring mRNA vaccine to balance innate/adaptive immune response. Trends Mol Med. 2020;26(3):311–23. 31699497 10.1016/j.molmed.2019.10.002

[40] Pulit-Penaloza JA, Scherbik SV, Brinton MA. Activation of Oas1a gene expression by type I IFN requires both STAT1 and STAT2 while only STAT2 is required for Oas1b activation. Virology. 2012;425(2):71–81. 22305621 10.1016/j.virol.2011.11.025 PMC3288655

[41] Karikó K, et al. Generating the optimal mRNA for therapy: HPLC purification eliminates immune activation and improves translation of nucleoside-modified, protein-encoding mRNA. Nucleic Acids Res. 2011;39(21):e142–142. 21890902 10.1093/nar/gkr695 PMC3241667

[42] Weissman D et al. *HPLC purification of in vitro transcribed long RNA.* Synthetic Messenger RNA and Cell Metabolism Modulation: Methods and Protocols, 2013: pp. 43–54. 10.1007/978-1-62703-260-5_3 23296926

[43] Baiersdörfer M, et al. A facile method for the removal of dsRNA contaminant from in vitro-transcribed mRNA. Mol Therapy-Nucleic Acids. 2019;15:26–35. 10.1016/j.omtn.2019.02.018 PMC6444222 30933724

[44] Shivalingam A, et al. Squaramides and ureas: a flexible approach to polymerase-compatible nucleic acid assembly. Angew Chem Int Ed. 2020;59(28):11416–22. 10.1002/anie.202000209 PMC7383975 32153132

[45] Muttach F, Muthmann N, Rentmeister A. Synthetic mRNA capping. Beilstein J Org Chem. 2017;13(1):2819–32. 30018667 10.3762/bjoc.13.274 PMC5753152

[46] Fuchs A-L, Neu A, Sprangers R. A general method for rapid and cost-efficient large-scale production of 5′ capped RNA. RNA. 2016;22(9):1454–66. 27368341 10.1261/rna.056614.116 PMC4986899

[47] Duan LJ, et al. Potentialities and challenges of mRNA vaccine in Cancer Immunotherapy. Front Immunol. 2022;13:923647. 35711457 10.3389/fimmu.2022.923647 PMC9196868

[48] Rydzik AM, et al. Synthesis and properties of mRNA cap analogs containing imidodiphosphate moiety—fairly mimicking natural cap structure, yet resistant to enzymatic hydrolysis. Bioorg Med Chem. 2012;20(5):1699–710. 22316555 10.1016/j.bmc.2012.01.013

[49] Schlake T, et al. Developing mRNA-vaccine technologies. RNA Biol. 2012;9(11):1319–30. 23064118 10.4161/rna.22269 PMC3597572

[50] Kumar P, et al. Inhibition of translation by IFIT family members is determined by their ability to interact selectively with the 5′-terminal regions of cap0-, cap1-and 5′ ppp-mRNAs. Nucleic Acids Res. 2014;42(5):3228–45. 24371270 10.1093/nar/gkt1321 PMC3950709

[51] Ringeard M, et al. FTSJ3 is an RNA 2′-O-methyltransferase recruited by HIV to avoid innate immune sensing. Nature. 2019;565(7740):500–4. 30626973 10.1038/s41586-018-0841-4

[52] Cao J, et al. Cap-dependent translation initiation factor, eIF4E, is the target for Ouabain-mediated inhibition of HIF-1α. Biochem Pharmacol. 2014;89(1):20–30. 24345331 10.1016/j.bcp.2013.12.002

[53] Whisenand JM, et al. Considerations for the Design and cGMP Manufacturing of mRNA therapeutics. San Diego, CA: TriLink BioTechnologies; 2017. p. 26.

[54] Al Fayez N et al. Recent Advancement in mRNA Vaccine Development and Applications. Pharmaceutics, 2023. 15(7). 10.3390/pharmaceutics15071972 PMC10384963 37514158

[55] von Niessen AGO, et al. Improving mRNA-based therapeutic gene delivery by expression-augmenting 3′ UTRs identified by cellular library screening. Mol Ther. 2019;27(4):824–36. 30638957 10.1016/j.ymthe.2018.12.011 PMC6453560

[56] Weissman D. mRNA transcript therapy. Expert Rev Vaccines. 2015;14(2):265–81. 25359562 10.1586/14760584.2015.973859

[57] Thess A, et al. Sequence-engineered mRNA without chemical nucleoside modifications enables an effective protein therapy in large animals. Mol Ther. 2015;23(9):1456–64. 26050989 10.1038/mt.2015.103 PMC4817881

[58] Vishweshwaraiah YL, Dokholyan NV. mRNA vaccines for cancer immunotherapy. Front Immunol. 2022;13:1029069. 36591226 10.3389/fimmu.2022.1029069 PMC9794995

[59] Mauro VP, Chappell SA. A critical analysis of codon optimization in human therapeutics. Trends Mol Med. 2014;20(11):604–13. 25263172 10.1016/j.molmed.2014.09.003 PMC4253638

[60] Proudfoot NJ. Ending the message: poly (A) signals then and now. Genes Dev. 2011;25(17):1770–82. 21896654 10.1101/gad.17268411 PMC3175714

[61] Lima SA, et al. Short poly (A) tails are a conserved feature of highly expressed genes. Nat Struct Mol Biol. 2017;24(12):1057–63. 29106412 10.1038/nsmb.3499 PMC5877826

[62] Li M, et al. Chapter seven - advances in mRNA vaccines. International Review of Cell and Molecular Biology. Academic; 2022. pp. 295–316. F. Aranda, P. Berraondo, and L. Galluzzi, Editors. 10.1016/bs.ircmb.2022.04.011 PMC9214710 36064266

[63] Karikó K, et al. Suppression of RNA recognition by toll-like receptors: the impact of Nucleoside modification and the Evolutionary Origin of RNA. Immunity. 2005;23(2):165–75. 16111635 10.1016/j.immuni.2005.06.008

[64] Kormann MS, et al. Expression of therapeutic proteins after delivery of chemically modified mRNA in mice. Nat Biotechnol. 2011;29(2):154–7. 21217696 10.1038/nbt.1733

[65] Pardi N, Weissman D. *Nucleoside modified mRNA vaccines for infectious diseases.* RNA vaccines: Methods and protocols, 2017: pp. 109–121. 10.1007/978-1-4939-6481-9_6 27987145

[66] Karikó K, et al. Incorporation of pseudouridine into mRNA yields superior nonimmunogenic vector with increased translational capacity and biological stability. Mol Ther. 2008;16(11):1833–40. 18797453 10.1038/mt.2008.200 PMC2775451

[67] Mei Y, Wang X. RNA modification in mRNA cancer vaccines. Clin Exp Med. 2023;23(6):1917–31. 36788153 10.1007/s10238-023-01020-5 PMC9928499

[68] Arango D, et al. Acetylation of cytidine in mRNA promotes translation efficiency. Cell. 2018;175(7):1872–86. e24. 30449621 10.1016/j.cell.2018.10.030 PMC6295233

[69] Wang H, et al. Mettl3-mediated mRNA m6A methylation promotes dendritic cell activation. Nat Commun. 2019;10(1):1898. 31015515 10.1038/s41467-019-09903-6 PMC6478715

[70] Yao R, Xie C, Xia X. Recent progress in mRNA cancer vaccines. Hum Vaccin Immunother. 2024;20(1):2307187. 38282471 10.1080/21645515.2024.2307187 PMC10826636

[71] Deng Z, et al. mRNA vaccines: the Dawn of a new era of Cancer Immunotherapy. Front Immunol. 2022;13:887125. 35720301 10.3389/fimmu.2022.887125 PMC9201022

[72] Qin S, et al. mRNA-based therapeutics: powerful and versatile tools to combat diseases. Signal Transduct Target Ther. 2022;7(1):166. 35597779 10.1038/s41392-022-01007-w PMC9123296

[73] Malla R, et al. mRNA vaccines and their delivery strategies: a journey from infectious diseases to cancer. Mol Ther. 2024;32(1):13–31. 37919901 10.1016/j.ymthe.2023.10.024 PMC10787123

[74] Pardi N, et al. mRNA vaccines—a new era in vaccinology. Nat Rev Drug Discovery. 2018;17(4):261–79. 29326426 10.1038/nrd.2017.243 PMC5906799

[75] Benteyn D, et al. mRNA-based dendritic cell vaccines. Expert Rev Vaccines. 2015;14(2):161–76. 25196947 10.1586/14760584.2014.957684

[76] Broderick KE, Humeau LM. Electroporation-enhanced delivery of nucleic acid vaccines. Expert Rev Vaccines. 2015;14(2):195–204. 25487734 10.1586/14760584.2015.990890

[77] Boczkowski D, et al. Dendritic cells pulsed with RNA are potent antigen-presenting cells in vitro and in vivo. J Exp Med. 1996;184(2):465–72. 8760800 10.1084/jem.184.2.465 PMC2192710

[78] Dannull J, et al. Enhancing the immunostimulatory function of dendritic cells by transfection with mRNA encoding OX40 ligand. Blood. 2005;105(8):3206–13. 15618466 10.1182/blood-2004-10-3944

[79] Aerts-Toegaert C, et al. CD83 expression on dendritic cells and T cells: correlation with effective immune responses. Eur J Immunol. 2007;37(3):686–95. 17301951 10.1002/eji.200636535

[80] Grünebach F, et al. Cotransfection of dendritic cells with RNA coding for HER-2/neu and 4-1BBL increases the induction of tumor antigen specific cytotoxic T lymphocytes. Cancer Gene Ther. 2005;12(9):749–56. 15877082 10.1038/sj.cgt.7700842

[81] Paston SJ, et al. Cancer vaccines, adjuvants, and Delivery systems. Front Immunol. 2021;12:627932. 33859638 10.3389/fimmu.2021.627932 PMC8042385

[82] Wilgenhof S, et al. A phase IB study on intravenous synthetic mRNA electroporated dendritic cell immunotherapy in pretreated advanced melanoma patients. Ann Oncol. 2013;24(10):2686–93. 23904461 10.1093/annonc/mdt245

[83] DeBenedette MA, et al. Potency of mature CD40L RNA electroporated dendritic cells correlates with IL-12 secretion by tracking multifunctional CD8+/CD28 + cytotoxic T-cell responses in vitro. J Immunother. 2011;34(1):45–57. 21150712 10.1097/CJI.0b013e3181fb651a

[84] Amin A, et al. Survival with AGS-003, an autologous dendritic cell–based immunotherapy, in combination with sunitinib in unfavorable risk patients with advanced renal cell carcinoma (RCC): phase 2 study results. J Immunother Cancer. 2015;3:1–13. 25901286 10.1186/s40425-015-0055-3 PMC4404644

[85] Mikhael J, et al. Treatment of multiple myeloma: ASCO and CCO Joint Clinical Practice Guideline. J Clin Oncol. 2019;37(14):1228–63. 30932732 10.1200/JCO.18.02096

[86] Pellat-Deceunynck C, et al. Isolation of human lymphocyte antigens class I-restricted cytotoxic T lymphocytes against autologous myeloma cells. Clin Cancer Res. 1999;5(3):705–9. 10100725

[87] Goodyear O, et al. CD8 + T cells specific for cancer germline gene antigens are found in many patients with multiple myeloma, and their frequency correlates with disease burden. Blood. 2005;106(13):4217–24. 16144804 10.1182/blood-2005-02-0563

[88] Chung DJ, et al. Langerhans dendritic cell vaccine bearing mRNA-encoded tumor antigens induces antimyeloma immunity after autotransplant. Blood Adv. 2022;6(5):1547–58. 35100339 10.1182/bloodadvances.2021005941 PMC8905697

[89] Selmi A, et al. Uptake of synthetic naked RNA by skin-resident dendritic cells via macropinocytosis allows antigen expression and induction of T-cell responses in mice. Cancer Immunol Immunother. 2016;65:1075–83. 27422115 10.1007/s00262-016-1869-7 PMC11028682

[90] Granstein RD, Ding W, Ozawa H. Induction of anti-tumor immunity with epidermal cells pulsed with tumor-derived RNA or intradermal administration of RNA. J Invest Dermatology. 2000;114(4):632–6. 10.1046/j.1523-1747.2000.00929.x 10733665

[91] Kreiter S, et al. Intranodal vaccination with naked antigen-encoding RNA elicits potent prophylactic and therapeutic antitumoral immunity. Cancer Res. 2010;70(22):9031–40. 21045153 10.1158/0008-5472.CAN-10-0699

[92] Bialkowski L, et al. Intralymphatic mRNA vaccine induces CD8 T-cell responses that inhibit the growth of mucosally located tumours. Sci Rep. 2016;6(1):22509. 26931556 10.1038/srep22509 PMC4773884

[93] Sahin U, et al. Personalized RNA mutanome vaccines mobilize poly-specific therapeutic immunity against cancer. Nature. 2017;547(7662):222–6. 28678784 10.1038/nature23003

[94] Lorentzen CL, et al. Clinical advances and ongoing trials on mRNA vaccines for cancer treatment. Lancet Oncol. 2022;23(10):e450–8. 36174631 10.1016/S1470-2045(22)00372-2 PMC9512276

[95] Morisaki T et al. Lymph nodes as Anti-tumor Immunotherapeutic Tools: Intranodal-Tumor-Specific Antigen-pulsed dendritic cell vaccine immunotherapy. Cancers (Basel), 2022. 14(10). 10.3390/cancers14102438 PMC9140043 35626042

[96] Ni L. Advances in mRNA-Based Cancer vaccines. Vaccines (Basel), 2023. 11(10). 10.3390/vaccines11101599 PMC10611059 37897001

[97] Zhou W-Z, et al. RNA melanoma vaccine: induction of antitumor immunity by human glycoprotein 100 mRNA immunization. Hum Gene Ther. 1999;10(16):2719–24. 10566900 10.1089/10430349950016762

[98] Kreiter S et al. *FLT3 ligand as a molecular adjuvant for naked RNA vaccines.* Synthetic mRNA: Production, Introduction Into Cells, and Physiological Consequences, 2016: pp. 163–175. 10.1007/978-1-4939-3625-0_11 27236799

[99] Kreiter S, et al. FLT3 ligand enhances the cancer therapeutic potency of naked RNA vaccines. Cancer Res. 2011;71(19):6132–42. 21816907 10.1158/0008-5472.CAN-11-0291

[100] Van Lint S, et al. Preclinical evaluation of TriMix and antigen mRNA-based antitumor therapy. Cancer Res. 2012;72(7):1661–71. 22337996 10.1158/0008-5472.CAN-11-2957

[101] Scheel B, et al. Therapeutic anti-tumor immunity triggered by injections of immunostimulating single‐stranded RNA. Eur J Immunol. 2006;36(10):2807–16. 17013976 10.1002/eji.200635910

[102] Van der Jeught K, et al. Intratumoral administration of mRNA encoding a fusokine consisting of IFN-β and the ectodomain of the TGF-β receptor II potentiates antitumor immunity. Oncotarget. 2014;5(20):10100. 25338019 10.18632/oncotarget.2463 PMC4259408

[103] Van Lint S, et al. Intratumoral delivery of TriMix mRNA results in T-cell activation by cross-presenting dendritic cells. Cancer Immunol Res. 2016;4(2):146–56. 26659303 10.1158/2326-6066.CIR-15-0163

[104] Wadhwa A, et al. Opportunities and challenges in the delivery of mRNA-based vaccines. Pharmaceutics. 2020;12(2):102. 32013049 10.3390/pharmaceutics12020102 PMC7076378

[105] Zhang C, et al. Advances in mRNA vaccines for infectious diseases. Front Immunol. 2019;10:594. 30972078 10.3389/fimmu.2019.00594 PMC6446947

[106] Zhang C, et al. Progress, challenges, and future of nanomedicine. Nano Today. 2020;35:101008.

[107] Zong Y, et al. Lipid nanoparticle (LNP) enables mRNA delivery for Cancer Therapy. Adv Mater. 2023;35(51):e2303261. 37196221 10.1002/adma.202303261

[108] Kon E, et al. Targeting cancer with mRNA-lipid nanoparticles: key considerations and future prospects. Nat Rev Clin Oncol. 2023;20(11):739–54. 37587254 10.1038/s41571-023-00811-9

[109] Kiaie SH, et al. Recent advances in mRNA-LNP therapeutics: immunological and pharmacological aspects. J Nanobiotechnol. 2022;20(1):276. 10.1186/s12951-022-01478-7 PMC9194786 35701851

[110] Hou X, et al. Lipid nanoparticles for mRNA delivery. Nat Rev Mater. 2021;6(12):1078–94. 34394960 10.1038/s41578-021-00358-0 PMC8353930

[111] Kon E, Elia U, Peer D. Principles for designing an optimal mRNA lipid nanoparticle vaccine. Curr Opin Biotechnol. 2022;73:329–36. 34715546 10.1016/j.copbio.2021.09.016 PMC8547895

[112] Thevenot J, et al. Steric stabilization of lipid/polymer particle assemblies by poly (ethylene glycol)-lipids. Biomacromolecules. 2007;8(11):3651–60. 17958441 10.1021/bm700753q

[113] Rojas LA, et al. Personalized RNA neoantigen vaccines stimulate T cells in pancreatic cancer. Nature. 2023;618(7963):144–50. 37165196 10.1038/s41586-023-06063-y PMC10171177

[114] Weber JS, et al. Individualised neoantigen therapy mRNA-4157 (V940) plus pembrolizumab versus pembrolizumab monotherapy in resected melanoma (KEYNOTE-942): a randomised, phase 2b study. Lancet. 2024;403(10427):632–44. 38246194 10.1016/S0140-6736(23)02268-7

[115] Jayaraman M, et al. Maximizing the potency of siRNA lipid nanoparticles for hepatic gene silencing in vivo. Angew Chem. 2012;124(34):8657–61. 10.1002/anie.201203263 PMC3470698 22782619

[116] Sabnis S, et al. A novel amino lipid series for mRNA delivery: improved endosomal escape and sustained pharmacology and safety in non-human primates. Mol Ther. 2018;26(6):1509–19. 29653760 10.1016/j.ymthe.2018.03.010 PMC5986714

[117] Hassett KJ, et al. Optimization of lipid nanoparticles for intramuscular administration of mRNA vaccines. Mol Therapy-Nucleic Acids. 2019;15:1–11. 10.1016/j.omtn.2019.01.013 PMC6383180 30785039

[118] Semple SC, et al. Rational design of cationic lipids for siRNA delivery. Nat Biotechnol. 2010;28(2):172–6. 20081866 10.1038/nbt.1602

[119] Miao L, et al. Synergistic lipid compositions for albumin receptor mediated delivery of mRNA to the liver. Nat Commun. 2020;11(1):2424. 32415122 10.1038/s41467-020-16248-y PMC7229004

[120] Akinc A, et al. A combinatorial library of lipid-like materials for delivery of RNAi therapeutics. Nat Biotechnol. 2008;26(5):561–9. 18438401 10.1038/nbt1402 PMC3014085

[121] Miao L, et al. Delivery of mRNA vaccines with heterocyclic lipids increases anti-tumor efficacy by STING-mediated immune cell activation. Nat Biotechnol. 2019;37(10):1174–85. 31570898 10.1038/s41587-019-0247-3

[122] Maier MA, et al. Biodegradable lipids enabling rapidly eliminated lipid nanoparticles for systemic delivery of RNAi therapeutics. Mol Ther. 2013;21(8):1570–8. 23799535 10.1038/mt.2013.124 PMC3734658

[123] Kauffman KJ, et al. Optimization of lipid nanoparticle formulations for mRNA delivery in vivo with fractional factorial and definitive screening designs. Nano Lett. 2015;15(11):7300–6. 26469188 10.1021/acs.nanolett.5b02497

[124] Dahlman JE, et al. Barcoded nanoparticles for high throughput in vivo discovery of targeted therapeutics. Proc Natl Acad Sci. 2017;114(8):2060–5. 28167778 10.1073/pnas.1620874114 PMC5338412

[125] Kowalski PS, et al. Delivering the messenger: advances in technologies for therapeutic mRNA delivery. Mol Ther. 2019;27(4):710–28. 30846391 10.1016/j.ymthe.2019.02.012 PMC6453548

[126] Zeng C, et al. *Formulation and delivery technologies for mRNA vaccines*, in *mRNA vaccines*. Springer; 2020. pp. 71–110. 10.1007/82_2020_217 PMC8195316 32483657

[127] Dahlman JE, et al. In vivo endothelial siRNA delivery using polymeric nanoparticles with low molecular weight. Nat Nanotechnol. 2014;9(8):648–55. 24813696 10.1038/nnano.2014.84 PMC4207430

[128] Khan OF, et al. Endothelial siRNA delivery in nonhuman primates using ionizable low–molecular weight polymeric nanoparticles. Sci Adv. 2018;4(6):eaar8409. 29963629 10.1126/sciadv.aar8409 PMC6021147

[129] Son S, et al. Sugar-nanocapsules imprinted with microbial molecular patterns for mRNA vaccination. Nano Lett. 2020;20(3):1499–509. 32023415 10.1021/acs.nanolett.9b03483 PMC7286077

[130] Chahal JS, et al. Dendrimer-RNA nanoparticles generate protective immunity against lethal Ebola, H1N1 influenza, and Toxoplasma Gondii challenges with a single dose. Proc Natl Acad Sci. 2016;113(29):pE4133–E4142. 10.1073/pnas.1600299113 PMC4961123 27382155

[131] Kaczmarek JC, et al. Polymer–lipid nanoparticles for systemic delivery of mRNA to the lungs. Angew Chem. 2016;128(44):14012–6. 10.1002/anie.201608450 PMC5279893 27690187

[132] Patel AK, et al. Inhaled nanoformulated mRNA polyplexes for protein production in lung epithelium. Adv Mater. 2019;31(8):1805116. 10.1002/adma.201805116 PMC7490222 30609147

[133] Kowalski PS, et al. Ionizable amino-polyesters synthesized via ring opening polymerization of tertiary amino‐alcohols for tissue selective mRNA delivery. Adv Mater. 2018;30(34):1801151. 10.1002/adma.201801151 PMC6320729 29975801

[134] Kaczmarek JC, et al. Optimization of a degradable polymer–lipid nanoparticle for potent systemic delivery of mRNA to the lung endothelium and immune cells. Nano Lett. 2018;18(10):6449–54. 30211557 10.1021/acs.nanolett.8b02917 PMC6415675

[135] McKinlay CJ, et al. Enhanced mRNA delivery into lymphocytes enabled by lipid-varied libraries of charge-altering releasable transporters. Proc Natl Acad Sci. 2018;115(26):E5859–66. 29891683 10.1073/pnas.1805358115 PMC6042134

[136] Haabeth OA, et al. mRNA vaccination with charge-altering releasable transporters elicits human T cell responses and cures established tumors in mice. Proc Natl Acad Sci. 2018;115(39):pE9153–E9161. 10.1073/pnas.1810002115 PMC6166849 30201728

[137] McKinlay CJ, et al. Charge-altering releasable transporters (CARTs) for the delivery and release of mRNA in living animals. Proc Natl Acad Sci. 2017;114(4):E448–56. 28069945 10.1073/pnas.1614193114 PMC5278438

[138] Scheel B, et al. Toll-like receptor‐dependent activation of several human blood cell types by protamine‐condensed mRNA. Eur J Immunol. 2005;35(5):1557–66. 15832293 10.1002/eji.200425656

[139] Sabari J, et al. Abstract B209: phase 1/2 study of mRNA vaccine therapy + durvalumab (durva) ± tremelimumab (treme) in patients with metastatic non-small cell lung cancer (NSCLC). Cancer Immunol Res. 2019;7(2Supplement):B209–209.

[140] Bell GD, et al. mRNA transfection by a xentry-protamine cell-penetrating peptide is enhanced by TLR antagonist E6446. PLoS ONE. 2018;13(7):e0201464. 30059522 10.1371/journal.pone.0201464 PMC6066245

[141] Myers R, et al. Oncolytic activities of approved mumps and measles vaccines for therapy of ovarian cancer. Cancer Gene Ther. 2005;12(7):593–9. 15746945 10.1038/sj.cgt.7700823

[142] Russell SJ, Peng KW. Measles virus for cancer therapy. Curr Top Microbiol Immunol. 2009;330:213–41. 19203112 10.1007/978-3-540-70617-5_11 PMC3926122

[143] Lou E. Oncolytic herpes viruses as a potential mechanism for cancer therapy. Acta Oncol. 2003;42(7):660–71. 14690152 10.1080/0284186031000518

[144] Bridle BW, et al. Vesicular stomatitis virus as a novel cancer vaccine vector to prime antitumor immunity amenable to rapid boosting with adenovirus. Mol Ther. 2009;17(10):1814–21. 19603003 10.1038/mt.2009.154 PMC2835010

[145] Larocca C, Schlom J. Viral vector-based therapeutic cancer vaccines. Cancer J. 2011;17(5):359–71. 21952287 10.1097/PPO.0b013e3182325e63 PMC3207353

[146] Chou JY, Mansfield BC. Recombinant AAV-directed gene therapy for type I glycogen storage diseases. Expert Opin Biol Ther. 2011;11(8):1011–24. 21504389 10.1517/14712598.2011.578067 PMC3126888

[147] Schott JW, et al. Viral and synthetic RNA vector technologies and applications. Mol Ther. 2016;24(9):1513–27. 27377044 10.1038/mt.2016.143 PMC5113109

[148] Rozovics JM, et al. Picornavirus modification of a host mRNA decay protein. MBio. 2012;3(6). 10.1128/mbio. 00431 – 12. 10.1128/mBio.00431-12 PMC3487778 23131833

[149] Vesin B, et al. An intranasal lentiviral booster reinforces the waning mRNA vaccine-induced SARS-CoV-2 immunity that it targets to lung mucosa. Mol Ther. 2022;30(9):2984–97. 35484842 10.1016/j.ymthe.2022.04.016 PMC9044714

[150] Ehrengruber MU, Schlesinger S, Lundstrom K. Alphaviruses: Semliki Forest virus and Sindbis virus vectors for gene transfer into neurons. Curr Protoc Neurosci. 2011;57(1):4221–42227. 10.1002/0471142301.ns0422s57 21971849

[151] Travieso T, et al. The use of viral vectors in vaccine development. npj Vaccines. 2022;7(1):75. 35787629 10.1038/s41541-022-00503-y PMC9253346

[152] Kaufman HL, et al. Local delivery of Vaccinia virus expressing multiple costimulatory molecules for the treatment of established tumors. Hum Gene Ther. 2006;17(2):239–44. 16454657 10.1089/hum.2006.17.239

[153] Moss B. Genetically engineered poxviruses for recombinant gene expression, vaccination, and safety. Proc Natl Acad Sci U S A. 1996;93(21):11341–8. 8876137 10.1073/pnas.93.21.11341 PMC38059

[154] Brown M, et al. Antigen gene transfer to cultured human dendritic cells using recombinant avipoxvirus vectors. Cancer Gene Ther. 1999;6(3):238–45. 10359209 10.1038/sj.cgt.7700014

[155] Drillien R, et al. Vaccinia virus-related events and phenotypic changes after infection of dendritic cells derived from human monocytes. Virology. 2000;268(2):471–81. 10704355 10.1006/viro.2000.0203

[156] Bonini C, et al. Targeting antigen in mature dendritic cells for simultaneous stimulation of CD4 + and CD8 + T cells. J Immunol. 2001;166(8):5250–7. 11290810 10.4049/jimmunol.166.8.5250

[157] Hodge JW, et al. Multiple costimulatory modalities enhance CTL avidity. J Immunol. 2005;174(10):5994–6004. 15879092 10.4049/jimmunol.174.10.5994 PMC1924685

[158] Yang S, Tsang KY, Schlom J. Induction of higher-avidity human CTLs by vector-mediated enhanced costimulation of antigen-presenting cells. Clin Cancer Res. 2005;11(15):5603–15. 16061879 10.1158/1078-0432.CCR-05-0670 PMC1351007

[159] Tezel A, et al. Topical delivery of anti-sense oligonucleotides using low-frequency sonophoresis. Pharm Res. 2004;21:2219–25. 15648253 10.1007/s11095-004-7674-6

[160] Eder JP, et al. A phase I trial of a recombinant vaccinia virus expressing prostate-specific antigen in advanced prostate cancer. Clin Cancer Res. 2000;6(5):1632–8. 10815880

[161] Marshall JL, et al. Phase I study in advanced cancer patients of a diversified prime-and-boost vaccination protocol using recombinant vaccinia virus and recombinant nonreplicating avipox virus to elicit anti-carcinoembryonic antigen immune responses. J Clin Oncol. 2000;18(23):3964–73. 11099326 10.1200/JCO.2000.18.23.3964

[162] Arlen PM, et al. Clinical safety of a viral vector based prostate cancer vaccine strategy. J Urol. 2007;178(4 Pt 1):1515–20. 17707059 10.1016/j.juro.2007.05.117

[163] Simon B, et al. Recombinant vaccines against infectious hematopoietic necrosis virus: production by the Caulobacter crescentus S-layer protein secretion system and evaluation in laboratory trials. Dis Aquat Organ. 2001;44(1):17–27. 11253870 10.3354/dao044017

[164] Herbst RS, et al. Predictive correlates of response to the anti-PD-L1 antibody MPDL3280A in cancer patients. Nature. 2014;515(7528):563–7. 25428504 10.1038/nature14011 PMC4836193

[165] Tumeh PC, et al. PD-1 blockade induces responses by inhibiting adaptive immune resistance. Nature. 2014;515(7528):568–71. 25428505 10.1038/nature13954 PMC4246418

[166] Ugel S, et al. Tumor-induced myeloid deviation: when myeloid-derived suppressor cells meet tumor-associated macrophages. J Clin Invest. 2015;125(9):3365–76. 26325033 10.1172/JCI80006 PMC4588310

[167] Mitchem JB, et al. Targeting tumor-infiltrating macrophages decreases tumor-initiating cells, relieves immunosuppression, and improves chemotherapeutic responses. Cancer Res. 2013;73(3):1128–41. 23221383 10.1158/0008-5472.CAN-12-2731 PMC3563931

[168] Ries CH, et al. Targeting tumor-associated macrophages with anti-CSF-1R antibody reveals a strategy for cancer therapy. Cancer Cell. 2014;25(6):846–59. 24898549 10.1016/j.ccr.2014.05.016

[169] Marigo I, et al. Tumor-induced tolerance and immune suppression depend on the C/EBPbeta transcription factor. Immunity. 2010;32(6):790–802. 20605485 10.1016/j.immuni.2010.05.010

[170] De Beuckelaer A, et al. Type I interferons interfere with the capacity of mRNA lipoplex vaccines to elicit cytolytic T cell responses. Mol Ther. 2016;24(11):2012–20. 27506450 10.1038/mt.2016.161 PMC5154477

[171] Pollard C, et al. Type I IFN counteracts the induction of antigen-specific immune responses by lipid-based delivery of mRNA vaccines. Mol Ther. 2013;21(1):251–9. 23011030 10.1038/mt.2012.202 PMC3538310

[172] Islam MA, et al. Adjuvant-pulsed mRNA vaccine nanoparticle for immunoprophylactic and therapeutic tumor suppression in mice. Biomaterials. 2021;266:120431. 33099060 10.1016/j.biomaterials.2020.120431 PMC7528902

[173] Papachristofilou A, et al. Phase ib evaluation of a self-adjuvanted protamine formulated mRNA-based active cancer immunotherapy, BI1361849 (CV9202), combined with local radiation treatment in patients with stage IV non-small cell lung cancer. J Immunother Cancer. 2019;7:1–14. 30736848 10.1186/s40425-019-0520-5 PMC6368815

[174] Luo M, et al. A STING-activating nanovaccine for cancer immunotherapy. Nat Nanotechnol. 2017;12(7):648–54. 28436963 10.1038/nnano.2017.52 PMC5500418

[175] Figlin RA, et al. Results of the ADAPT phase 3 study of rocapuldencel-T in combination with sunitinib as first-line therapy in patients with metastatic renal cell carcinoma. Clin Cancer Res. 2020;26(10):2327–36. 32034074 10.1158/1078-0432.CCR-19-2427

[176] Bonehill A, et al. Enhancing the T-cell stimulatory capacity of human dendritic cells by co-electroporation with CD40L, CD70 and constitutively active TLR4 encoding mRNA. Mol Ther. 2008;16(6):1170–80. 18431362 10.1038/mt.2008.77

[177] De Keersmaecker B et al. TriMix and tumor antigen mRNA electroporated dendritic cell vaccination plus ipilimumab: link between T-cell activation and clinical responses in advanced melanoma. J Immunother Cancer, 2020. 8(1). 10.1136/jitc-2019-000329 PMC7057443 32114500

[178] Naka T, et al. Tumor vaccine therapy against recrudescent tumor using dendritic cells simultaneously transfected with tumor RNA and granulocyte macrophage colony-stimulating factor RNA. Cancer Sci. 2008;99(2):407–13. 18271939 10.1111/j.1349-7006.2007.00698.x PMC11158764

[179] Bontkes H, et al. Dendritic cells transfected with interleukin-12 and tumor-associated antigen messenger RNA induce high avidity cytotoxic T cells. Gene Ther. 2007;14(4):366–75. 17036057 10.1038/sj.gt.3302874

[180] Minkis K, et al. Type 2 bias of T cells expanded from the blood of melanoma patients switched to type 1 by IL-12p70 mRNA–transfected dendritic cells. Cancer Res. 2008;68(22):9441–50. 19010919 10.1158/0008-5472.CAN-08-0900 PMC2628575

[181] Van den Bergh J, et al. Transpresentation of interleukin-15 by IL-15/IL-15Rα mRNA-engineered human dendritic cells boosts antitumoral natural killer cell activity. Oncotarget. 2015;6(42):44123. 26675759 10.18632/oncotarget.6536 PMC4792546

[182] Van den Bergh J et al. *Characterization of interleukin-15-transpresenting dendritic cells for clinical use.* Journal of Immunology Research, 2017. 2017. 10.1155/2017/1975902 PMC5530419 28785596

[183] Liu X, et al. mRNA Cancer vaccines: construction and boosting strategies. ACS Nano. 2023;17(20):19550–80. 37819640 10.1021/acsnano.3c05635

[184] Shijie M, et al. Immunotherapeutic treatment of lung cancer and bone metastasis with a mPLA/mRNA tumor vaccine. Acta Biomater. 2023;169:489–99. 37536492 10.1016/j.actbio.2023.07.059

[185] Kauffman KJ, Webber MJ, Anderson DG. Materials for non-viral intracellular delivery of messenger RNA therapeutics. J Controlled Release. 2016;240:227–34. 10.1016/j.jconrel.2015.12.032 26718856

[186] Guan S, Rosenecker J. Nanotechnologies in delivery of mRNA therapeutics using nonviral vector-based delivery systems. Gene Ther. 2017;24(3):133–43. 28094775 10.1038/gt.2017.5

[187] Reichmuth AM, et al. mRNA vaccine delivery using lipid nanoparticles. Therapeutic Delivery. 2016;7(5):319–34. 27075952 10.4155/tde-2016-0006 PMC5439223

[188] Midoux P, Pichon C. Lipid-based mRNA vaccine delivery systems. Expert Rev Vaccines. 2015;14(2):221–34. 25540984 10.1586/14760584.2015.986104

[189] Pardi N, et al. Expression kinetics of nucleoside-modified mRNA delivered in lipid nanoparticles to mice by various routes. J Controlled Release. 2015;217:345–51. 10.1016/j.jconrel.2015.08.007 PMC4624045 26264835

[190] Kranz LM, et al. Systemic RNA delivery to dendritic cells exploits antiviral defence for cancer immunotherapy. Nature. 2016;534(7607):396–401. 27281205 10.1038/nature18300

[191] Kübler H, et al. Self-adjuvanted mRNA vaccination in advanced prostate cancer patients: a first-in-man phase I/IIa study. J Immunother Cancer. 2015;3(1):1–14. 26082837 10.1186/s40425-015-0068-y PMC4468959

[192] Qiu P, et al. Gene gun delivery of mRNA in situ results in efficient transgene expression and genetic immunization. Gene Ther. 1996;3(3):262–8. 8646558

[193] Johansson DX, et al. Intradermal electroporation of naked replicon RNA elicits strong immune responses. PLoS ONE. 2012;7(1):e29732. 22238645 10.1371/journal.pone.0029732 PMC3251598

[194] Ols S, et al. Route of vaccine administration alters antigen trafficking but not innate or adaptive immunity. Cell Rep. 2020;30(12):3964–71. e7. 32209459 10.1016/j.celrep.2020.02.111 PMC7198771

[195] Tam HH, et al. Sustained antigen availability during germinal center initiation enhances antibody responses to vaccination. Proc Natl Acad Sci U S A. 2016;113(43):E6639–48. 27702895 10.1073/pnas.1606050113 PMC5086995

[196] Liang SL, Quirk D, Zhou A. RNase L: its biological roles and regulation. IUBMB Life. 2006;58(9):508–14. 17002978 10.1080/15216540600838232

[197] Liang F, et al. Dissociation of skeletal muscle for flow cytometric characterization of immune cells in macaques. J Immunol Methods. 2015;425:69–78. 26099800 10.1016/j.jim.2015.06.011 PMC4604051

[198] Vormehr M et al. *Mutanome engineered RNA immunotherapy: towards patient-centered tumor vaccination.* Journal of Immunology Research, 2015. 2015. 10.1155/2015/595363 PMC4710911 26844233

[199] Copur MS. *Messenger RNA Vaccines: Beckoning of a New Era in Cancer Immunotherapy.* Oncology (08909091), 2021. 35(4). 10.46883/ONC.2021.3504.0198 33893760

[200] Bol KF, et al. Intranodal vaccination with mRNA-optimized dendritic cells in metastatic melanoma patients. Oncoimmunology. 2015;4(8):e1019197. 26405571 10.1080/2162402X.2015.1019197 PMC4570143

[201] Ols S, Loré K. Imaging the early fate of mRNA vaccines. Nat Biomedical Eng. 2019;3(5):331–2. 10.1038/s41551-019-0399-y 31073175

[202] Broos K et al. Particle-mediated intravenous delivery of antigen mRNA results in strong antigen-specific T-cell responses despite the induction of type I interferon. Mol Therapy-Nucleic Acids, 2016. 5. 10.1038/mtna.2016.38 PMC5022130 27327138

[203] Bauer T, et al. Abstract CT210: a phase I, open-label, multicenter, dose escalation study of mRNA-2752, a lipid nanoparticle encapsulating mRNAs encoding human OX40L, IL-23, and IL-36γ, for intratumoral injection alone and in combination with immune checkpoint blockade. Cancer Res. 2019;79(13Supplement):CT210–210.

[204] Wang E, Aifantis I. RNA splicing and cancer. Trends cancer. 2020;6(8):631–44. 32434734 10.1016/j.trecan.2020.04.011

[205] Wang Y, et al. The roles of alternative splicing in tumor-immune cell interactions. Curr Cancer Drug Targets. 2020;20(10):729–40. 32560607 10.2174/1568009620666200619123725 PMC8388066

[206] Efremova M, et al. Neoantigens generated by individual mutations and their role in cancer immunity and immunotherapy. Front Immunol. 2017;8:1679. 29234329 10.3389/fimmu.2017.01679 PMC5712389

[207] Cafri G, et al. mRNA vaccine-induced neoantigen-specific T cell immunity in patients with gastrointestinal cancer. J Clin Invest. 2020;130(11):5976–88. 33016924 10.1172/JCI134915 PMC7598064

[208] Blass E, Ott PA. Advances in the development of personalized neoantigen-based therapeutic cancer vaccines. Nat Reviews Clin Oncol. 2021;18(4):215–29. 10.1038/s41571-020-00460-2 PMC7816749 33473220

[209] Xie N, et al. Neoantigens: promising targets for cancer therapy. Signal Transduct Target Therapy. 2023;8(1):9. 10.1038/s41392-022-01270-x PMC9816309 36604431

[210] Li W-H, Li Y-M. Chemical strategies to Boost Cancer vaccines. Chem Rev. 2020;120(20):11420–78. 32914967 10.1021/acs.chemrev.9b00833

[211] Pritchard AL, et al. Exome sequencing to Predict neoantigens in Melanoma. Cancer Immunol Res. 2015;3(9):992–8. 26048577 10.1158/2326-6066.CIR-15-0088

[212] Rosenthal R, et al. Neoantigen-directed immune escape in lung cancer evolution. Nature. 2019;567(7749):479–85. 30894752 10.1038/s41586-019-1032-7 PMC6954100

[213] Tran E, et al. T-Cell transfer Therapy Targeting Mutant KRAS in Cancer. N Engl J Med. 2016;375(23):2255–62. 27959684 10.1056/NEJMoa1609279 PMC5178827

[214] Kyte J, et al. Phase I/II trial of melanoma therapy with dendritic cells transfected with autologous tumor-mRNA. Cancer Gene Ther. 2006;13(10):905–18. 16710345 10.1038/sj.cgt.7700961

[215] Kyte JA, et al. Immune response and long-term clinical outcome in advanced melanoma patients vaccinated with tumor-mRNA-transfected dendritic cells. Oncoimmunology. 2016;5(11):e1232237. 27999747 10.1080/2162402X.2016.1232237 PMC5139630

[216] Kantoff PW, et al. Sipuleucel-T immunotherapy for castration-resistant prostate cancer. N Engl J Med. 2010;363(5):411–22. 20818862 10.1056/NEJMoa1001294

[217] Batich KA, et al. Long-term survival in glioblastoma with cytomegalovirus pp65-targeted vaccination. Clin Cancer Res. 2017;23(8):1898–909. 28411277 10.1158/1078-0432.CCR-16-2057 PMC5559300

[218] Rittig SM, et al. Long-term survival correlates with immunological responses in renal cell carcinoma patients treated with mRNA-based immunotherapy. Oncoimmunology. 2016;5(5):e1108511. 27467913 10.1080/2162402X.2015.1108511 PMC4910748

[219] Sahin U, et al. An RNA vaccine drives immunity in checkpoint-inhibitor-treated melanoma. Nature. 2020;585(7823):107–12. 32728218 10.1038/s41586-020-2537-9

